# PPARγ Agonists Improve Survival and Neurocognitive Outcomes in Experimental Cerebral Malaria and Induce Neuroprotective Pathways in Human Malaria

**DOI:** 10.1371/journal.ppat.1003980

**Published:** 2014-03-06

**Authors:** Lena Serghides, Chloe R. McDonald, Ziyue Lu, Miriam Friedel, Cheryl Cui, Keith T. Ho, Howard T. J. Mount, John G. Sled, Kevin C. Kain

**Affiliations:** 1 Toronto General Research Institute, University Health Network, Toronto, Ontario, Canada; 2 SA Rotman Laboratories, Sandra Rotman Centre for Global Health, University Health Network, Toronto, Ontario, Canada; 3 Women's College Research Institute, Women's College Hospital, Toronto, Ontario, Canada; 4 Tropical Disease Unit, Division of Infectious Diseases, Department of Medicine, University of Toronto, Toronto, Ontario, Canada; 5 Mouse Imaging Centre, Hospital for Sick Children, Toronto, Ontario, Canada; 6 Department of Physiology, University of Toronto, Toronto, Ontario, Canada; 7 Department of Psychiatry, Department of Medicine, University of Toronto, Toronto, Ontario, Canada; 8 Department of Medical Biophysics, University of Toronto, Toronto, Ontario, Canada; London School of Hygiene and Tropical Medicine, United Kingdom

## Abstract

Cerebral malaria (CM) is associated with a high mortality rate, and long-term neurocognitive impairment in approximately one third of survivors. Adjunctive therapies that modify the pathophysiological processes involved in CM may improve outcome over anti-malarial therapy alone. PPARγ agonists have been reported to have immunomodulatory effects in a variety of disease models. Here we report that adjunctive therapy with PPARγ agonists improved survival and long-term neurocognitive outcomes in the *Plasmodium berghei* ANKA experimental model of CM. Compared to anti-malarial therapy alone, PPARγ adjunctive therapy administered to mice at the onset of CM signs, was associated with reduced endothelial activation, and enhanced expression of the anti-oxidant enzymes SOD-1 and catalase and the neurotrophic factors brain derived neurotrophic factor (BDNF) and nerve growth factor (NGF) in the brains of infected mice. Two months following infection, mice that were treated with anti-malarials alone demonstrated cognitive dysfunction, while mice that received PPARγ adjunctive therapy were completely protected from neurocognitive impairment and from PbA-infection induced brain atrophy. In humans with *P. falciparum* malaria, PPARγ therapy was associated with reduced endothelial activation and with induction of neuroprotective pathways, such as BDNF. These findings provide insight into mechanisms conferring improved survival and preventing neurocognitive injury in CM, and support the evaluation of PPARγ agonists in human CM.

## Author Summary

Cerebral malaria (CM) is a severe complication of *Plasmodium falciparum* infection that is associated with long-term neurocognitive impairment in about a third of survivors even when optimal anti-malarial therapy is used. Since both the parasite and the host immune response to infection play a role in the development of CM, adjunctive therapies that modulate the host response, given in conjunction with anti-parasitic therapy, may improve survival and prevent neurocognitive injury. Here we examine the effects of PPARγ agonists on neurocongitive injury using a mouse model of CM. We demonstrate that PPARγ agonists, when administered with anti-malarials, protected mice from developing brain atrophy and neurocognitive impairment. This was associated with induction of anti-oxidant and neuroprotective pathways in the brains of infected mice. We also observed the same neuroprotective pathways induced in patients with falciparum malaria that received PPARγ adjunctive therapy. Our findings suggest that PPARγ agonists may be valuable in the treatment and prevention of CM-induced neurocognitive injury, and support the testing of PPARγ agonists in patients with CM.

## Introduction

Cerebral malaria (CM) is a severe complication of *Plasmodium falciparum* infection that is associated with high mortality rates despite potent anti-anti-malarial therapy [1]–[2]. Adjunctive therapies, aimed at modifying the pathophysiological processes of malaria infection have been pursued as a way to improve outcome in CM, albeit with limited success to date [3].

CM is characterized by seizures and coma in the presence of parasitemia and in the absence of any other recognized cause of coma. While it was long believed that the majority of children surviving CM were left neurologically intact, recent studies have challenged this assumption and provided evidence that many CM survivors have long-term cognitive and neurological deficits [4]–[6].

Both parasite and host determinants contribute to the onset and neurological outcome of CM. Host innate immune responses to infection combined with sequestration of parasitized erythrocytes (PEs) in the microvasculature of the brain, result in dysregulated inflammation, endothelial activation and dysfunction, microvascular occlusion, vascular leak, and ultimately loss of blood-brain barrier (BBB) function and integrity [7]. Sequestered PEs, perfusion abnormalities, hemorrhages, edema, local tissue hypoxia and ischemia, and focal disruptions of the BBB are common fundoscopic and autopsy findings in CM patients [8]–[9]. Oxidative stress and axonal injury in the vicinity of brain hemorrhages and in areas of vascular occlusion are observed in CM and may contribute to neurological dysfunction pre-mortem and in CM survivors [10]. In experimental models of CM (ECM), intravital microscopy studies have revealed that neurological signs in ECM are associated with dysfunction of the neuroimmunological BBB, including vascular leak from post-capillary venules [11].

Peroxisome proliferators-activated receptor gamma (PPARγ) is a member of the family of nuclear hormone receptors that function as ligand-activated transcription factors [12]. PPARγ agonists are reported to have anti-inflammatory and anti-oxidant properties [13], and may also possess neuroprotective properties mediated in part by promoting neuron repair mechanisms [14]–[15]. We have previously shown that PPARγ agonists decrease human monocyte inflammatory responses to *P. falciparum* PEs *in vitro*, and reduce systemic inflammation and improve survival *in vivo* in the ECM model [16]. These pre-clinical observations were extended and confirmed in a randomized double-blind placebo controlled trial in Thai patients with *P. falciparum* malaria. In this clinical trial adjunctive therapy with PPARγ agonists was associated with significantly faster parasite clearance time and reduced systemic inflammatory responses to infection [17]. Based on the above observations and putative role of PPARγ activation in neuroprotection, we tested the hypothesis that PPARγ agonists would enhance endothelial quiescence and BBB integrity, and activate neuroprotective pathways in mice and humans. Here we report that PPARγ agonist therapy resulted in improved survival and long-term neurocognitive performance in the ECM model and was associated with the induction of neuroprotective pathways, such as brain derived neurotrophic factor (BDNF), in both *P. berghei* ANKA-infected mice and humans with *P. falciparum* malaria.

## Results

### Adjunctive therapy with PPARγ agonists improves survival in ECM

**Figure 1:**
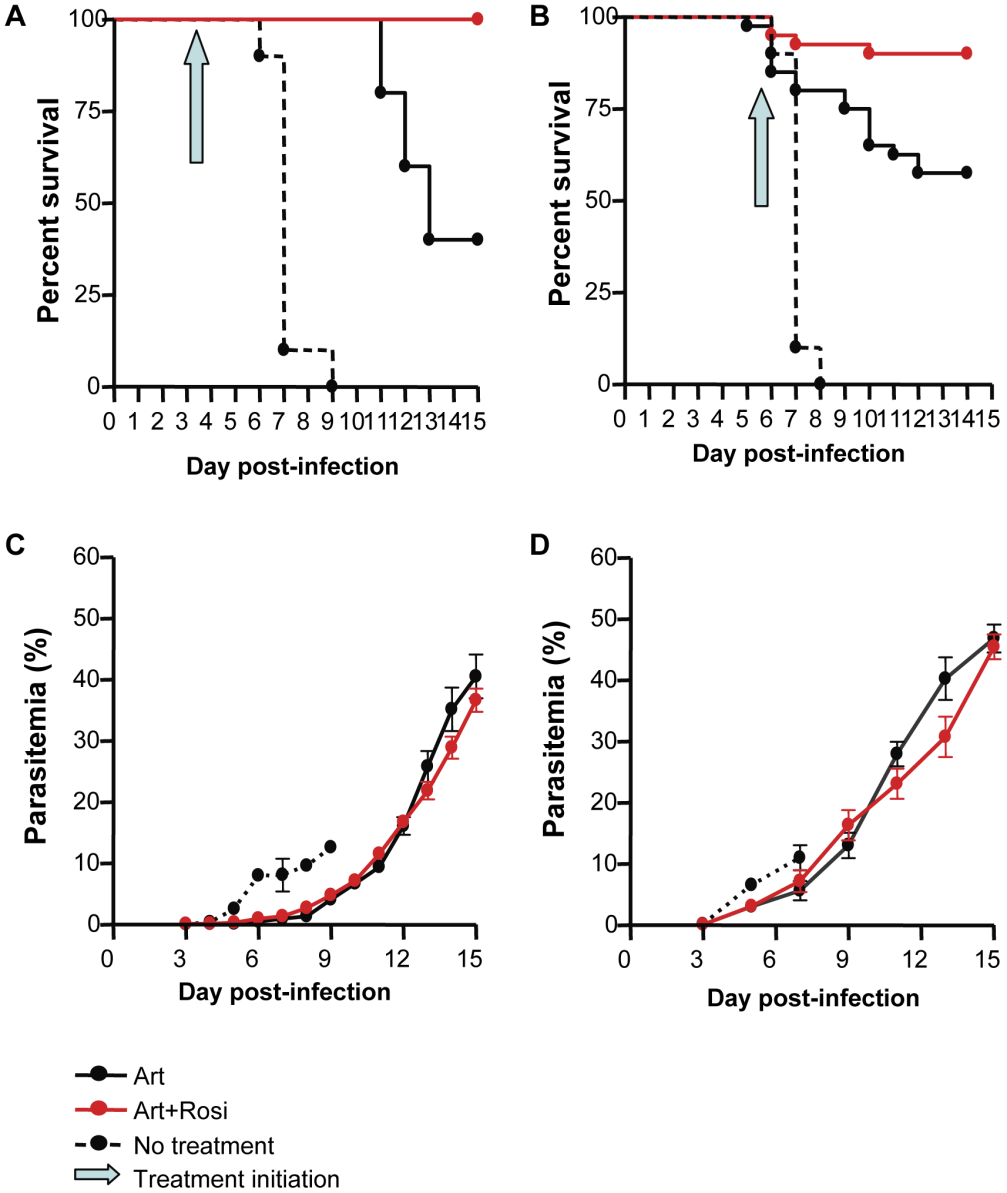
Rosiglitazone adjunctive therapy improves survival in ECM. Survival curves (A and B) and parasitemia (C and D) for mice infected with PbA and receiving, no treatment (dashed line), 10 mg/kg artesunate plus saline (solid black line), or 10 mg/kg artesunate plus 2.5 mg/kg rosiglitazone (red line). The blue arrow indicates treatment initiation (day 3 post-infection for A and C, and onset of CM for B and D). In (A), p = 0.0042 for artesunate vs. artesunate + rosiglitazone; p<0.0001 for no treatment vs. artesunate + rosiglitazone, and for no treatment vs. artesunate, by Logrank test, N = 10/group. In (B), p = 0.0012 for artesunate vs. artesunate + rosiglitazone; p<0.0001 for no treatment vs. artesunate + rosiglitazone, and for no treatment vs. artesunate, by Logrank test, N = 40 for the artesunate and artesunate + rosiglitazone groups, N = 10 for the no treatment group. (C–D) Parasitemia curves did not differ significantly between artesunate and artesunate + rosiglitazone groups. Abbreviations: Art, artesunate; Rosi, rosiglitazone.

We have previously shown that mice treated prophylactically with rosiglitazone are protected from developing CM and have significantly higher survival rates than control mice [16]. To model clinically relevant scenarios we designed treatment protocols in the ECM model whereby *Plasmodium berghei* ANKA (PbA)-infected mice were treated with a combination of artesunate (10 mg/kg), and a PPARγ agonist (rosiglitazone; 2.5 mg/kg) or saline as a control starting on day 3 post-infection, when parasitemia becomes detectable peripherally, or when mice displayed neurological symptoms and began to die from CM (between day 5 and 6 of infection) (see Figure S1 for treatment protocols). Starting treatment on day 3 post-infection resulted in 100% survival in the rosiglitazone-treated group compared to a 40% survival in the group receiving artesunate alone (Figure 1A, p = 0.0042). All untreated mice succumbed to their infection. Starting treatment at the onset of CM resulted in a 90% survival in the rosiglitazone-treated group compared to 57.5% survival in the group receiving artesunate alone (Figure 1B, p = 0.0012). Similar survival benefit was observed with pioglitazone (a PPARγ agonist belonging to the same class of drugs as rosiglitazone). Pioglitazone (20 mg/kg) adjunctive therapy commencing at the onset of CM signs resulted in an 85% survival compared to 45.5% survival in the group receiving artesunate alone (p = 0.0093, by Logrank test, n = 20/group). Parasitemia did not significantly differ between the treatment groups (Figure 1C–D). All mice that survived the CM window went on to develop hyperparasitemia.

### PPARγ therapy reduces endothelial activation and enhances blood brain barrier integrity

**Figure 2:**
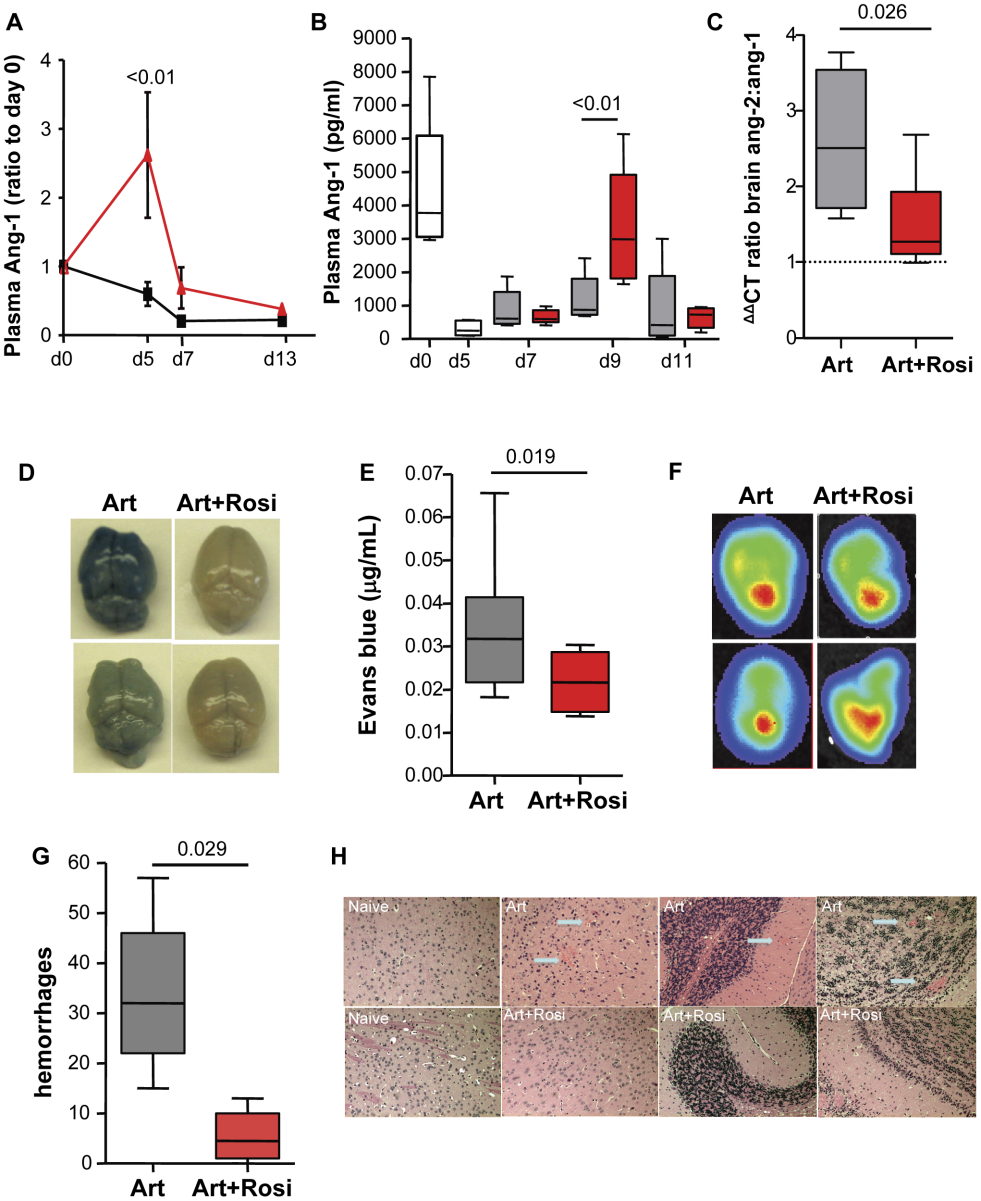
Rosiglitazone adjunctive therapy helps maintain peripheral angiopoietin-1 levels, and protects from blood brain barrier breakdown. (A) Mice infected with PbA were treated with artesunate plus saline (black line) or artesunate plus rosiglitazone (red line) starting on day 3 post-infection. Blood was collected serially on day 0, 5, 7, and 13 and angiopoietin-1 (Ang-1) levels were tested. Ang-1 levels were normalised to d0 values. Data were analysed by two-way ANOVA with Bonferroni post-test, N = 6/group. (B-C) Mice infected with PbA were treated with artesunate plus saline (Art, grey bars), or artesunate plus rosiglitazone (Art+Rosi, red bars) beginning at the onset of CM signs. Plasma levels of Ang1 are shown in (B). The white bars indicate ang-1 levels prior to treatment. The ratio of ang-2 to ang-1 mRNA expression in the brain of mice on day 7 post-infection is shown in (C). The dashed line indicates median values for uninfected mice. Data shown are medians with range. Statistical comparisons by Kruskal-Wallis with Dunn's post-test in B and Mann Whitney test in C, N = 6/group, one of two independent experiments. (D–H) Mice infected with PbA or luciferase-expressing PbA (PbGFP-LUC(con)) were treated with either artesunate plus saline (Art) or artesunate plus rosiglitazone (Art+Rosi) beginning on day 3 post-infection. (D–E) On day 10 of infection parasitemia matched mice were injected with Evans blue to assess vascular leak in the brain. Photographs of representative brains from artesunate (on the left) and artesunate plus rosiglitazone (on the right) treated mice are shown in (D), and quantification of Evans blue in the brains is shown in (E). The dashed line is the median Evans blue value for uninfected mice. All comparisons by Mann Whitney test, N = 10/group. (F) Parasite accumulation was assessed by bioluminescence detection of mice injected with luciferin. Parasite accumulation was similar between groups. (G) Brain hemorrhages were assessed blindly on formalin-fixed brain sections from parasitemia-matched mice collected on day 10 post-infection. Comparisons by Mann Whitney, N = 5/group. Data shown are medians with range. (H) Representative images of H&E stained brain sections. Blue arrows indicate hemorrhages.

Endothelial activation and dysfunction leading to loss of vascular integrity are thought to be central to the pathophysiology of CM. The angiopoietin-Tie-2 system has been identified as a key regulator of vascular integrity, and plays a role in disease progression and outcome in malaria infection [18]. The interaction between angiopoietin-1 (Ang-1) and its receptor Tie-2 promotes endothelial quiescence and inhibits vascular leakage, while Ang-2 antagonizes the Ang-1-Tie-2 interaction and promotes endothelial activation. We investigated whether rosiglitazone adjunctive therapy has an impact on angiopoietin levels in ECM. Mice receiving adjunctive rosiglitazone therapy (starting either on day 3 post-infection or at the onset of neurological signs) had significantly higher circulating levels of Ang-1 (Figure 2A and B, and Figure S2), consistent with less endothelial activation compared to mice receiving artesunate alone. Compared to uninfected mice, Ang-1 levels were significantly lower in infected mice treated with artesunate alone, while Ang-1 levels were maintained in mice that received rosiglitazone adjunctive therapy. Using real time quantitative PCR analysis of brain homogenates we observed significantly higher Ang-1 mRNA levels (Figure S3A), a trend towards lower Ang-2 mRNA levels (Figure S3B), and a significantly lower Ang-2 to Ang-1 mRNA ratio in mice treated with rosiglitazone compared to those treated with artesunate alone (Figure 2C and Figure S3C). Our data suggest that rosiglitazone adjunctive therapy induces a shift in the Ang-2 to Ang-1 balance towards a more quiescent endothelium.

We extended these observations to investigate whether this was also associated with enhanced BBB integrity and reduced vascular leakage during ECM. Mice receiving rosiglitazone had significantly reduced levels of vascular leak (as assessed by Evans blue) and preserved BBB function compared to mice treated with artesunate alone (Figure 2D–E), despite having similar peripheral parasitemia levels and similar parasite accumulation in the brain (Figure 2F) (as assessed by bioluminescence imaging of mice infected with luciferase expressing PbA) [19]. Compared to mice treated with artesunate alone, mice receiving rosiglitazone also had significantly fewer brain hemorrhages (Figure 2G and H), providing additional evidence of enhanced BBB integrity and neuroprotection.

To further examine whether the elevated levels of Ang-1 observed in rosiglitazone-treated mice directly contributed to improved survival and neuroprotection we infected mice that were either sufficient for Ang-1 or had one Ang-1 allele deleted (Ang-1^del^, the kind gift of Dr. SE Quaggin [20]) with PbA and treated them with artesunate plus either rosiglitazone or saline as a control starting on day 5.5 post-infection. Ang-1^del^ mice produce 30-50% the Ang-1 levels of Ang-1 sufficient mice (Figure S4). Rosiglitazone significantly improved survival and reduced disease severity (as assessed by a modified rapid murine coma and behaviour scale (RMCBS) [21]) only in the Ang-1 sufficient mice, but not in the Ang-1^del^ mice (Figure S4), supporting a direct role for Ang-1 in mediating, at least partly, the observed protective effects of rosiglitazone.

### PPARγ therapy increases expression of the anti-oxidant enzymes SOD-1 and catalase in the brains of PbA-infected mice

**Figure 3:**
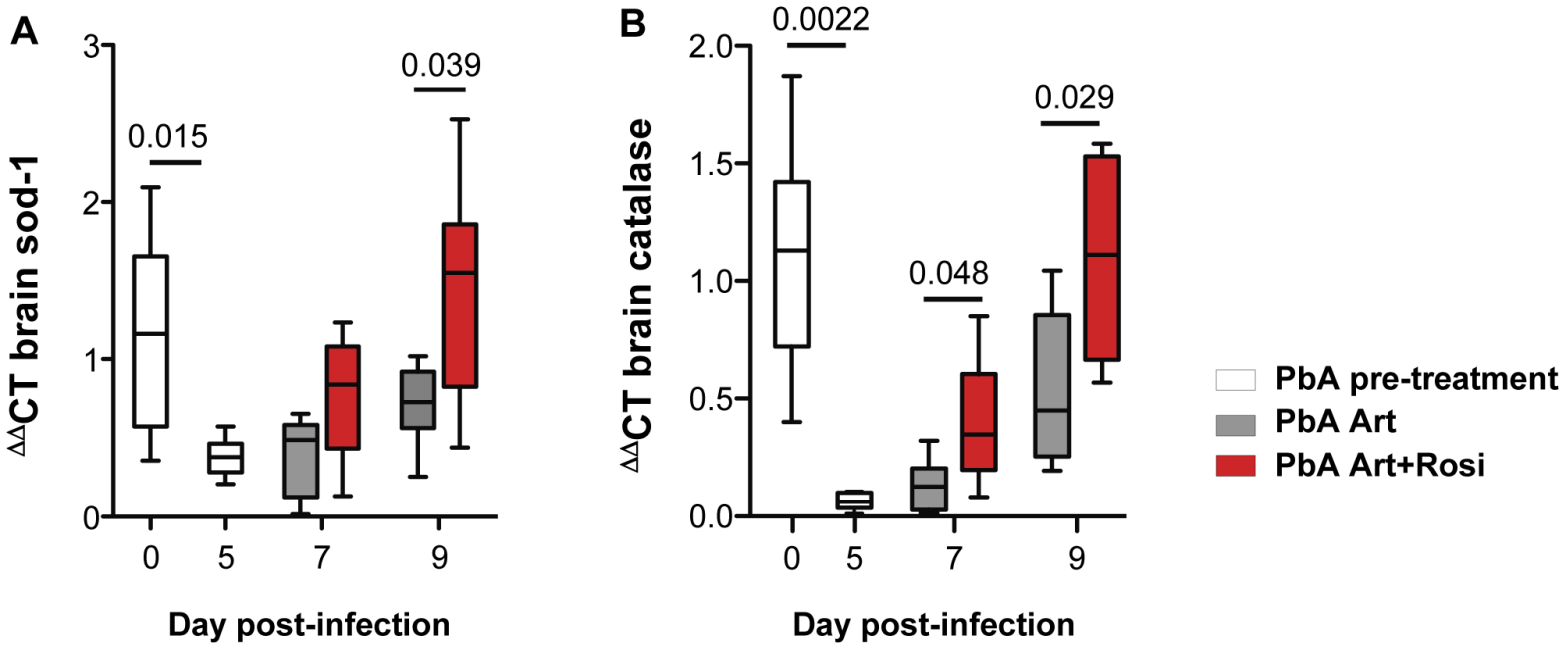
Rosiglitazone adjunctive therapy increases brain expression of the anti-oxidant enzymes SOD-1 and catalase. Mice infected with PbA were treated with artesunate plus saline (grey bars), or artesunate plus rosiglitazone (red bars) starting at the onset of CM signs. Expression of SOD-1 (A) and catalase (B) mRNA was assessed in brain homogenates collected from uninfected mice (day 0), infected mice prior to the initiation of therapy (day 5), and infected mice following treatment initiation (on day 7 and 9 post-infection). Data were analysed by one-way ANOVA with Bonferroni's multiple comparison test, N = 6 per group.

Malaria infection is associated with release of free heme that can trigger oxidative stress and tissue injury. Oxidative stress and axonal damage are thought to contribute to neuronal injury and dysfunction in CM patients [10]. Increased oxidative stress has also been observed in the brains of mice with ECM [22]. Upregulation of endogenous anti-oxidant enzymes can help maintain redox balance and protect neurons from injury. In agreement with its putative neuroprotective role, anti-oxidant activity is decreased in patients who develop CM and in mice susceptible to ECM [22]–[23]. PbA infection was associated with a significant decrease in brain mRNA levels of the anti-oxidant enzymes SOD-1 and catalase (Figure 3A–B, day 0 vs. day 5). Compared to mice receiving artesunate alone, addition of rosiglitazone adjunctive therapy resulted in significantly increased levels of SOD-1 and catalase expression and full recovery to pre-infection levels by day 9 post-infection (Figure 3A–B). We did not observed a significant difference in HO-1 levels between treatment groups (Figure S5).

In summary, rosiglitazone treatment resulted in higher brain expression of SOD-1 and catalase, compared to artesunate treatment alone.

### PPARγ agonists increase expression of neurotrophic factors BDNF and NGF in the brains of PbA-infected mice

**Figure 4:**
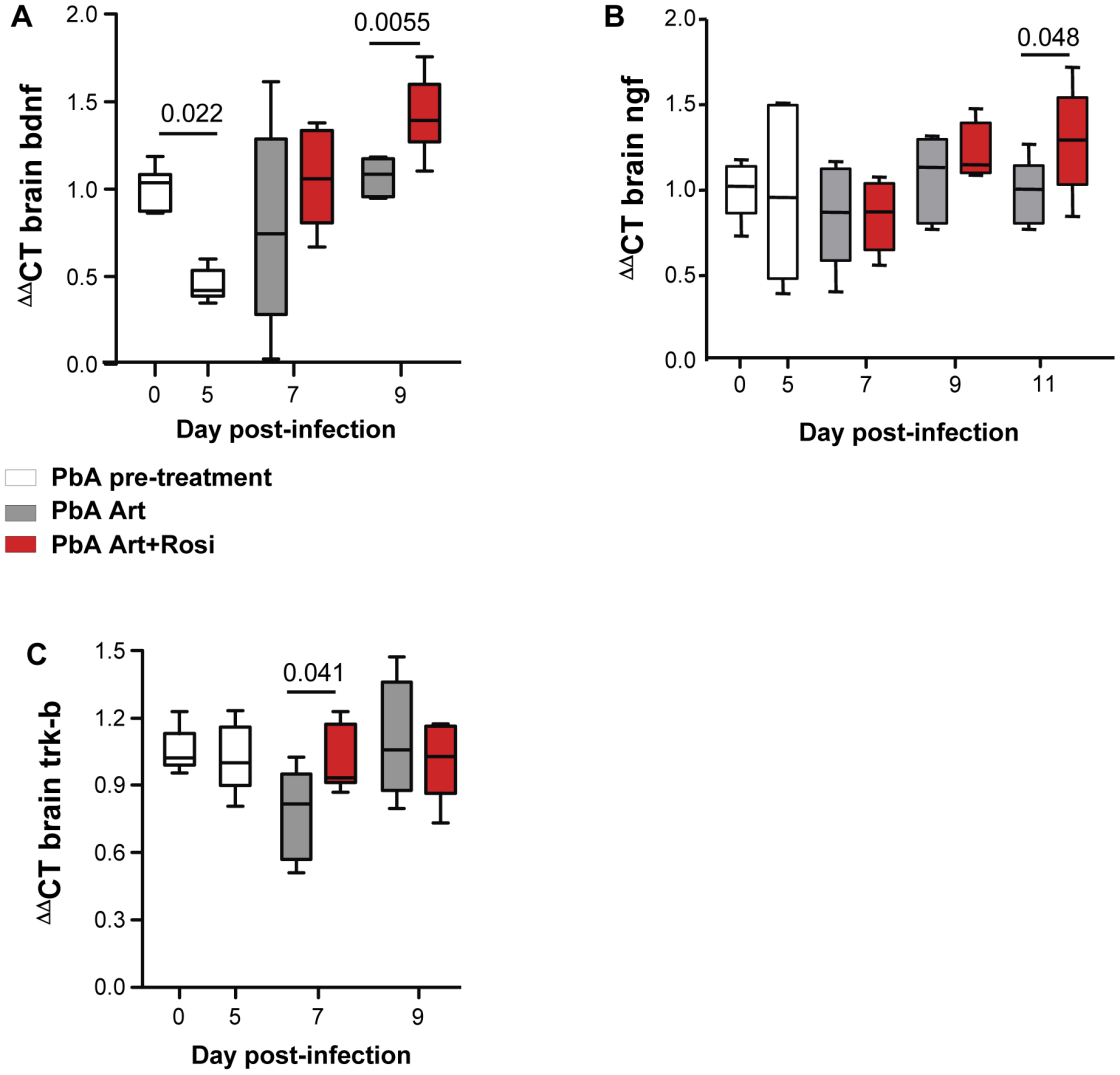
Rosiglitazone adjunctive therapy increases expression of neurotrophic factors in the brains of malaria-infected mice. (A–C) Mice infected with PbA were treated with artesunate plus saline (grey bars), or artesunate plus rosiglitazone (red bars) starting at the onset of CM signs. Expression of BDNF (A), NGF (B), and the BDNF receptor Trk-B (C) mRNA was assessed in brain homogenates collected from uninfected mice (day 0), infected mice prior to the initiation of therapy (day 5), and infected mice following treatment initiation (on day 7 and 9 post-infection). All comparisons by one-way ANOVA with Bonferroni's multiple comparison tests.

Neurotrophic factors, such as BDNF and nerve growth factor (NGF) play an important neuroprotective role [24]. Decreased BDNF mRNA expression in the brain has been correlated with increased CM severity in PbA-infected mice [25]. In agreement with previous observations, we observed a 55% decrease in BDNF mRNA expression in the brains of PbA-infected mice at the onset of neurological signs (Figure 4A day 0 vs. day 5). Mice receiving rosiglitazone adjunctive therapy had higher levels of BDNF compared to mice receiving artesunate alone (Figure 4A). BDNF expression was significantly higher in the rosiglitazone group on day 9 post-infection. Although NGF levels were not affected by PbA infection, rosiglitazone treatment was also associated with higher levels of NGF expression on day 11 post-infection (Figure 4B).

The synaptic effects of BDNF are mediated by its receptor Trk-B [26]. Trk-B expression in the brain was preserved in mice receiving rosiglitazone, while it decreased on day 7 post-infection in mice receiving artesunate alone (Figure 4C).

Thus, rosiglitazone adjunctive therapy was associated with both elevated levels of BDNF and NGF expression and maintenance of Trk-B expression in the brains of mice infected with malaria.

### PPARγ adjunctive therapy protects mice from cognitive and motor impairment

Long-term neurocognitive impairment is observed in children surviving CM. Mice, drug-cured following the onset of ECM, also demonstrate neurocognitive impairment [22], [27]. Our data thus far indicated that rosiglitazone adjunctive therapy is associated with improved survival in ECM but it is unclear if this is at the cost of increased neurocognitive injury in ECM survivors. Alternatively our observations of preserved BBB integrity and induction of CNS anti-oxidant enzymes and neurotrophic factors suggest that PPARγ-treatment might confer neuroprotection in ECM. To test this latter hypothesis, we drug-cured mice at the onset of CM signs with a combination of artesunate and mefloquine, with or without rosiglitazone adjunctive therapy for a total of 7 days (see Figure S1 for dosing regimen). Mice remained parasite-free following treatment for the entire testing period. Uninfected mice that received the same drug treatment regimens were used as controls. Mice were evaluated using a battery of standardized neurocognitive tests of learning, memory, exploratory behaviour, anxiety, and motor performance, starting 2 months following the completion of curative anti-malarial therapy. Compared to infected mice cured with artesunate/mefloquine alone, we observed significant improvements in neurocognitive performance in mice that received rosiglitazone adjunctive therapy.

**Figure 5:**
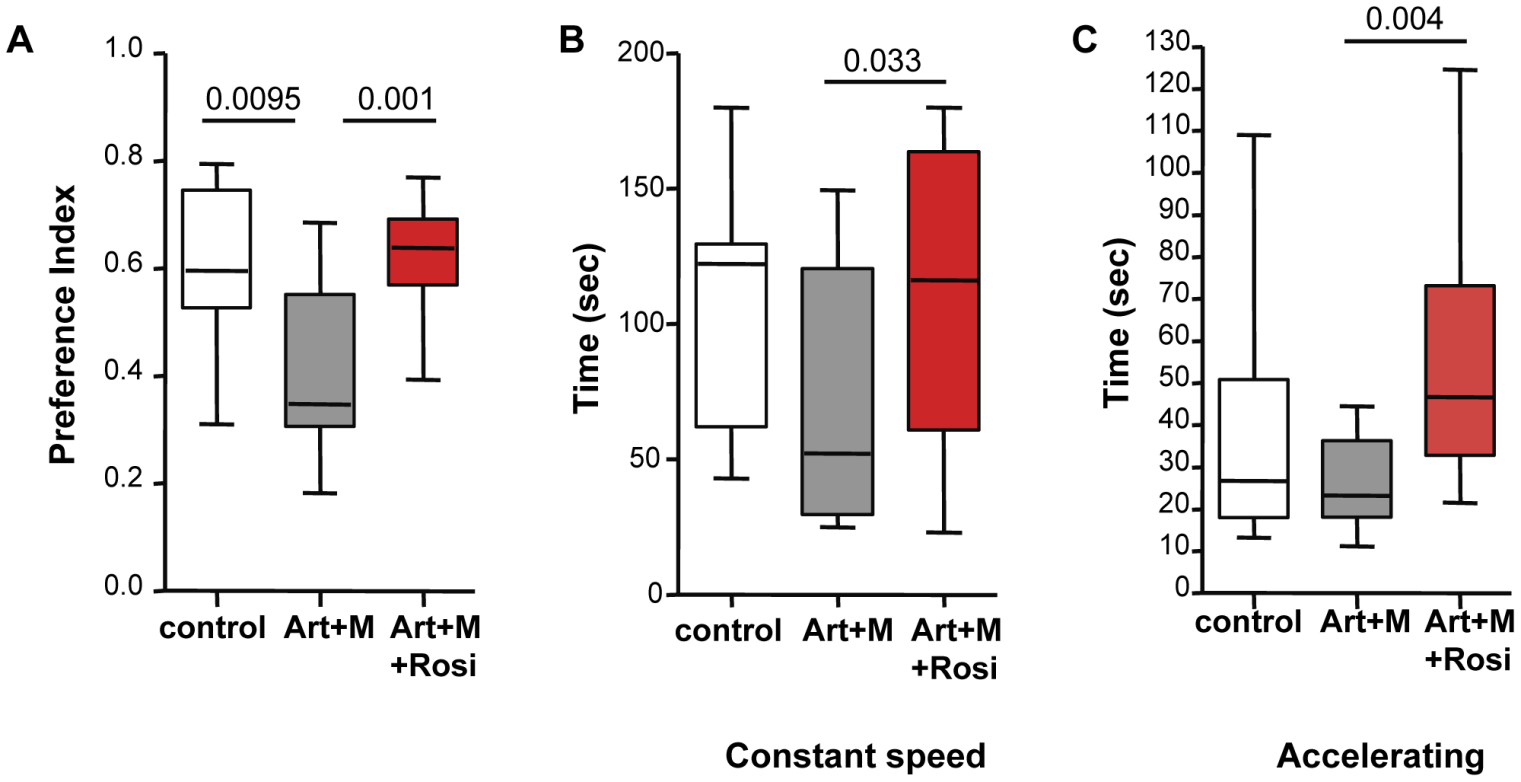
Mice treated with rosiglitazone adjunctive therapy perform better in the novel object recognition and rotarod tests. Mice infected with PbA were drug-cured at the onset of CM with artesunate/mefloquine plus saline (grey bars), or plus rosiglitazone (red bars). Drug-treated uninfected mice were used as controls (white bars). Mice remained parasite-free throughout testing. Testing was performed 2 months following completion of treatment. Data for the novel object recognition test (NOR) are shown in (A). The preference index indicates the preference for exploring the novel object over the familiar one. Data for the constant speed rotarod test are shown in (B) and for the accelerating rotarod test in (C). The average latency (measured in seconds) to fall off the rod apparatus for 5 trials is shown. All comparisons by one-way ANOVA with Boneferroni's multiple comparison tests.

The novel object recognition (NOR) test is a test of non-spatial learning and memory and is based on the innate tendency of rodents to preferentially explore novel objects over familiar ones. PbA-infected mice treated with artesunate/mefloquine had significantly lower memory (preference index) scores compared to uninfected controls for the novel object following a 3-hour retention interval (Figure 5A). In contrast, PbA-infected mice that received rosiglitazone adjunctive therapy had preference index scores similar to uninfected controls, and significantly better than mice that were treated with artesunate/mefloquine alone (Figure 5A). Differences in preference scores could not be attributed to differences in mobility or total exploration time between the groups (Figure S6).

To assess motor performance we conducted both a constant and an accelerating speed rotarod test. In both tests PbA-infected mice that received rosiglitazone adjunctive therapy performed significantly better than PbA-infected mice treated with artesunate/mefloquine alone (Figure 5B–C), suggesting better motor coordination and balance in the rosiglitazone-treated group. In both tests the PbA-infected mice treated with artesunate/mefloquine alone showed impaired performance compared with uninfected controls, however the difference between the groups did not reach statistical significance (p = 0.063 for constant rotarod test, and p = 0.12 for accelerating rotarod test).

We did not observe any differences between groups in the open field test, which assesses exploratory and locomotor behaviour, in the tail suspension test, which assesses anxiety and depression, or in the contextual fear-conditioning test, which assesses associative learning (Figure S7).

These data suggest that CM is associated with deficits in memory and motor skills, and that rosiglitazone adjunctive therapy conferred protection to these ECM-associated neurocognitive deficits.

### PPARγ therapy protects mice from PbA-induced brain atrophy

**Figure 6:**
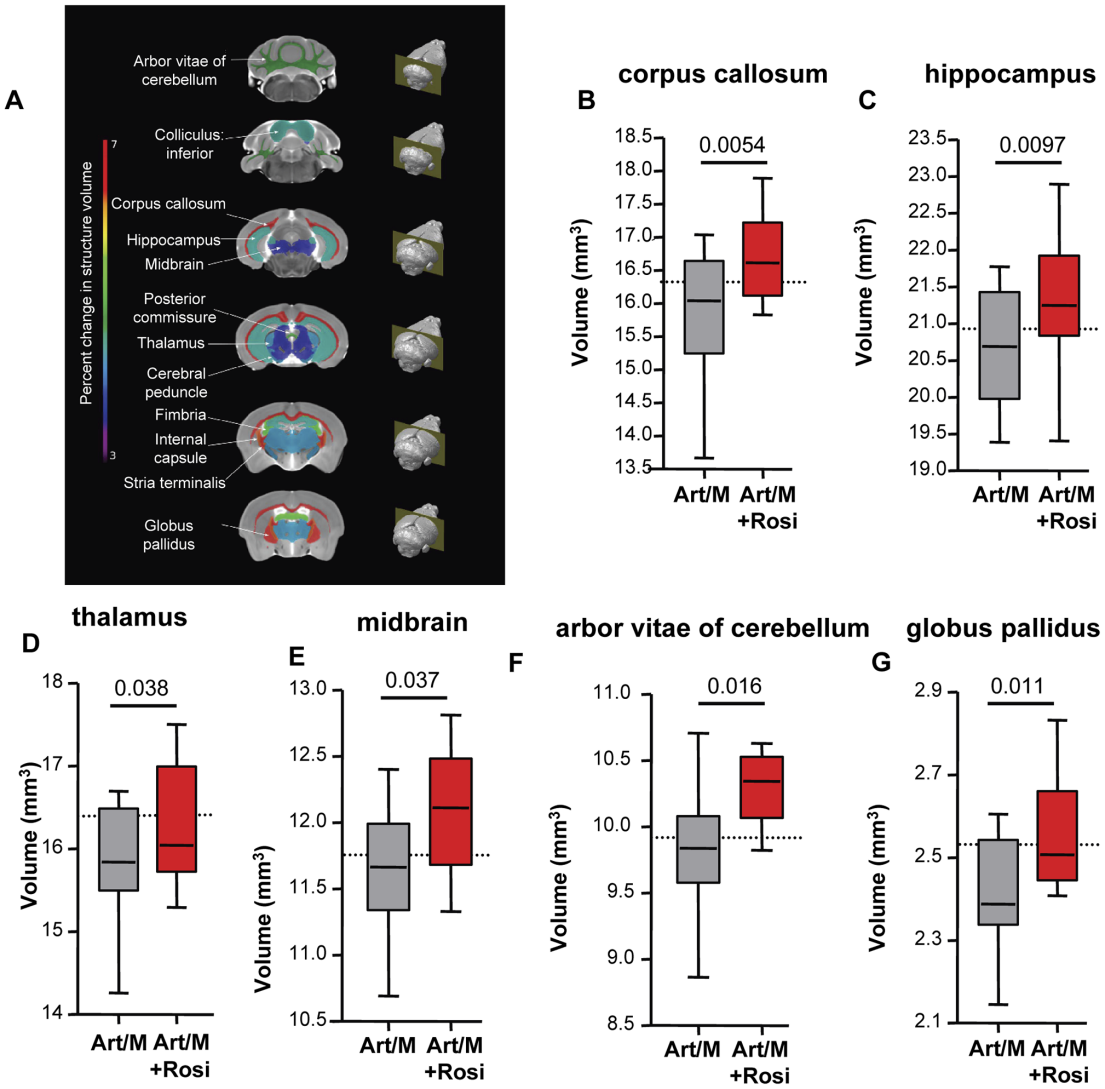
Rosiglitazone adjunctive therapy protects mice from malaria-induced brain atrophy. Following completion of all behavioural testing mouse brains were scanned using magnetic resonance imaging (MRI). Image registration and volumetric analysis of the brain volume of 62 distinct regions were performed. Linear regression analysis was used to identify areas that differed significantly between the mice treated with artesunate/mefloquine plus saline (grey bars), and the mice treated with artesunate/mefloquine plus rosiglitazone (red bars). (A) Identified brain regions are shown. Percentage change in brain structure volume is indicated by colour (see rainbow bar on the left). Significant differences were observed in (B) the corpus callosum, (C) the hippocampus, (D) the thalamus, (E) the midbrain, (F) the arbour vitae of the cerebellum, and (G) the globus pallidus of the basal ganglia. N = 13 for artesunate/mefloquine, N = 10 for artesunate/mefloquine + rosiglitazone. The median volume for uninfected mice is shown as a dashed line (N = 10 for uninfected control). Additional brain regions are shown in Figure S8.

Specific brain regions can undergo structural plastic changes in response to demands or injury, such as learning a new task, or response to and recovery from a serious infection [28]. CT and MRI assessment of children during acute CM and of CM survivors with neurological sequelae have demonstrated brain structural abnormalities including focal and multifocal atrophy [29]–[30]. We next performed high-resolution MRI studies to determine if the behavioural phenotypes we observed correlated with morphological changes in the CNS. Following completion of all behavioral testing, mice were perfused and brain tissue was fixed for MRI. Volumetric analysis was performed on total brain volume and 62 distinct brain regions outlined based on a previously established mouse brain atlas [31]–[32]. We observed significant differences in 12 brain regions between mice that received anti-malarials alone and mice that received rosiglitazone adjunctive therapy (Figure 6A and Figure S8). In all cases the brain regions had a preserved (larger) volume in the rosiglitazone-treated mice. Notably, we observed significant differences in the corpus callosum, hippocampus, and thalamus (Figure 6B–D). These areas are involved in new memory formation and detection of novel events and stimuli. Atrophy in these areas may relate to the impaired responses observed in the NOR test with mice treated with anti-malarial alone. We also observed significant differences in the midbrain, the arbor vitae of the cerebellum, and the globus pallidus of the basal ganglia (Figure 6E–G). These are regions that play a critical role in motor coordination and balance, and corresponding atrophy in these areas may be related to the impaired rotarod test performance observed in mice treated with anti-malarials alone.

In summary, we observed that rosiglitazone adjunctive therapy was associated with protection from brain injury/atrophy in brain regions relevant to memory and motor functions.

### Rosiglitazone adjunctive therapy in patients with *P. falciparum* malaria is associated with a lower Ang-2 to Ang-1 ratio and higher levels of BDNF

**Figure 7:**
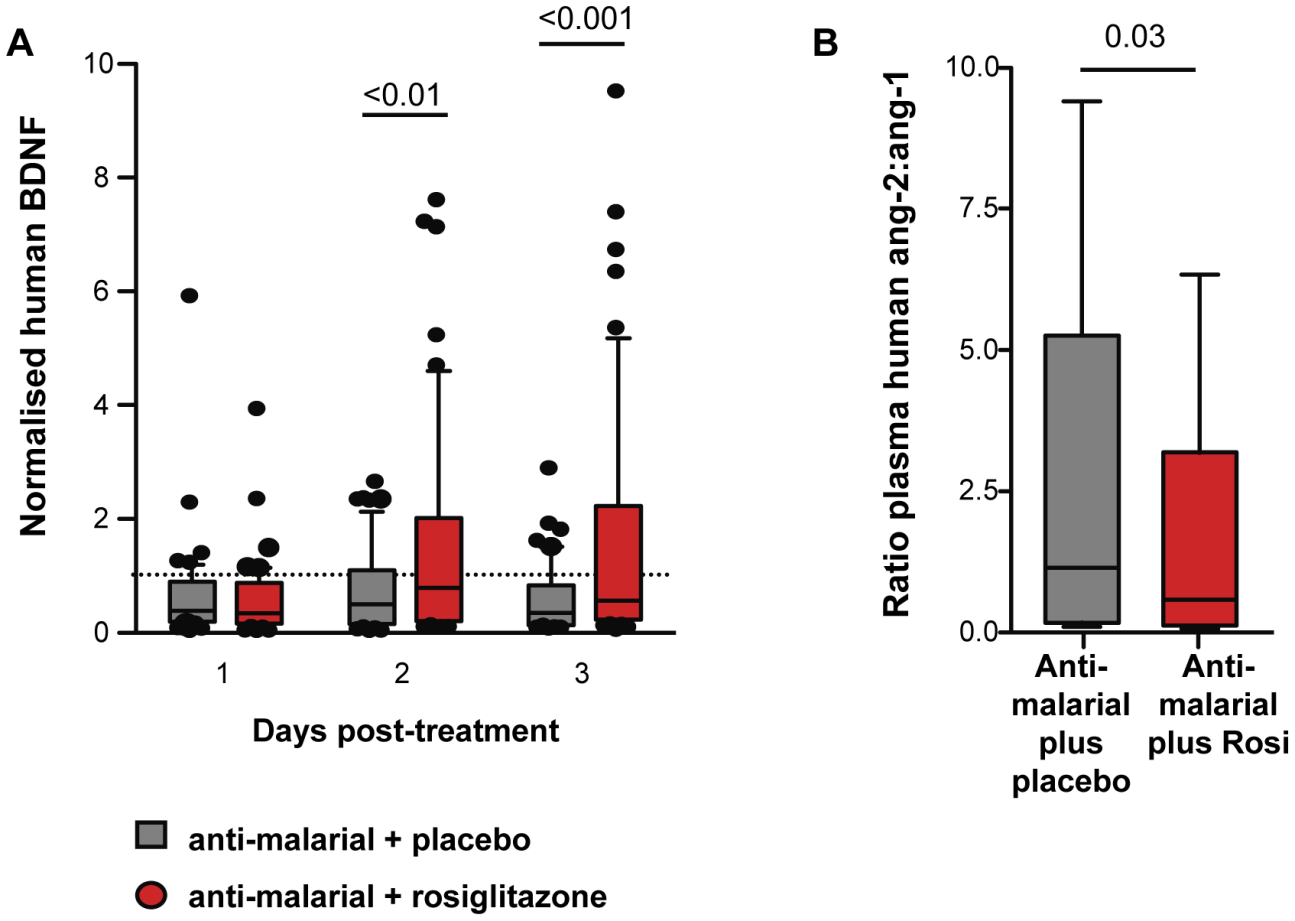
Rosiglitazone adjunctive therapy increased BDNF and lowered the angiopoietin-2:angiopoietin-1 ratio in patient with falciparum malaria. BDNF, Ang-1, and Ang-2 levels were determined in plasma collected from patients with uncomplicated malaria randomised to atovaquone proguanil plus either rosiglitazone or placebo. (A) BDNF levels were normalized to levels measured prior to treatment initiation. Normalised BDNF levels for day 1-3 post-treatment initiation are shown. Comparisons between placebo vs. rosiglitazone by Kruskal-Wallis test with Dunn's post-test, N = 52/group. Comparison between baseline vs. treatment by Wilcoxon Signed Rank test: p<0.001, p = 0.0024, and p<0.0001 for placebo baseline vs. placebo day 1, day 2, and day 3 respectively; and p<0.0001, p = 0.84, and p = 0.94 for rosiglitazone baseline vs. day 1, day 2, and day 3 respectively. (B) The ratio of Ang-2 to Ang-1 in plasma assessed 3 days post-treatment initiation. Data shown are medians with range. All comparisons by Mann Whitney test. N = 67/group.

To extend our findings to a relevant human population we examined the levels of Ang-1, Ang-2, and BDNF in plasma banked from patients that had participated in a randomized double-blind placebo controlled trial of rosiglitazone plus atovaquone-proguanil (AP) versus AP plus placebo for the treatment of uncomplicated *P. falciparum* malaria acquired on the Thai-Burmese border [17]. At the time of our trial recruitment (2004–2005) AP was an accepted 1^st^ line agent for the treatment of uncomplicated mefloquine-resistant malaria prevalent in the study area. Patients were randomly assigned to receive either placebo or rosiglitazone (4 mg twice daily) for 3 days. The primary end points of this trial were safety and tolerability of rosiglitazone administration as adjunctive therapy of uncomplicated *P. falciparum* malaria. Secondary endpoints were 50% and 90% parasite clearance time (PCT, the time required for patients to reduce their parasite burden by 50% and 90%). Rosiglitazone was found to be safe and well tolerated, and patients receiving rosiglitazone had significantly shorter 50% and 90% PCT, [rosiglitazone vs. placebo: 19.0 h vs. 24.6 h (p = 0.029) for 50% PCT; and 30.9 h vs. 40.4 h (p = 0.004) for 90% PCT [17]. We also detected significantly lower levels of inflammatory biomarkers IL-6 and MCP-1 in the rosiglitazone arm [17]. Patients receiving rosiglitazone had significantly higher levels of BDNF starting on day 2 post-treatment initiation (Figure 7A), and a significantly lower Ang-2 to Ang-1 ratio on day 3 post-treatment initiation compared to those receiving placebo (Figure 7B).

## Discussion

Cerebral malaria is associated with high fatality rates and long-term neurodisabilities despite optimal anti-malarial therapy. There is a need for adjunctive therapies to reduce mortality and to improve neurocognitive outcome in survivors. Here we report that adjunctive therapy with the PPARγ agonist rosiglitazone was associated with enhanced survival, and complete protection from neurocognitive impairment and acute brain injury and subsequent brain atrophy in mice infected with PbA. Rosiglitazone improved outcome in part by reducing endothelial activation, increasing BBB integrity, and upregulating expression levels of the anti-oxidant enzymes SOD-1 and catalase, and the neurotrophic factors BDNF and NGF in the brains of infected mice. We further report that rosiglitazone adjunctive therapy was also associated with reduced endothelial activation, as evidenced by a lower Ang-2 to Ang-1 ratio, and enhanced levels of the neuroprotective factor BDNF in patients with *P. falciparum* malaria.

Recent studies have demonstrated epilepsy, persistent attention and memory problems, developmental delays in language and gross motor function, and attention deficit hyperactivity disorder (ADHD)-like behavior in ∼one-third of children who survive CM [4]–[6], [33]–[35]. Mice, drug-cured following the onset of ECM, also demonstrate neurocognitive deficits [27]. We observed that rosiglitazone, administered concomitantly with anti-malarial therapy beginning at the onset of ECM, completely protected mice from long-term neurocognitive impairment, specifically impairment in learning, memory, attention, and motor coordination. Mice that received rosiglitazone performed as well as uninfected mice and significantly better than infected mice that received anti-malarials alone.

High resolution MRI analysis for morphological CNS changes in mice drug-cured at the onset of ECM revealed significant volumetric differences between mice that received rosiglitazone and those that did not. The observed volumetric differences in the CNS correlated well with the behavioural phenotype observed in mice surviving ECM. Rosiglitazone treatment was associated with less atrophy in brain areas implicated in memory and spatial learning, specifically the corpus callosum, hippocampus, and thalamus, and in areas implicated in motor performance, specifically the cerebellum, basal ganglia, and midbrain. Injuries to these brain areas generate symptoms that are often seen in CM survivors, including deficits in learning, memory, and executive functions, problems with verbal fluency, impaired motor function, and ADHD-like symptoms [36]–[40]. Of note, our ECM data mirror MRI and CT findings observed in human CM. Changes in brain morphology, including changes in brain volume, abnormal T2 intensity, and diffusion-weighted MRI abnormalities, have been reported in acute CM and in the majority of CM survivors with neurological sequelae [29]–[30], [41]. Abnormal T2 signal intensity and DWI (diffusion weighted imaging) abnormalities in the basal ganglia was identified in 84% of children with CM, and over 49% of children showed such abnormalities in the thalamus, corpus callosum, and cerebellum.

Rosiglitazone therapy was associated with the induction of several potentially neuroprotective and neurorestorative pathways, including regulation of BDNF. As previously reported [25], [42], we observed reduced BDNF levels in the brains of PbA-infected mice. While anti-malarial treatment was associated with some increase in BDNF levels, mice that received rosiglitazone adjunctive therapy had significantly higher levels of BDNF and NGF expression in their brain, compared to mice receiving anti-malarials alone. Decreases in BDNF levels have been associated with alterations in neuronal maintenance and regeneration, structural abnormalities in the brain, and reduced neuronal plasticity resulting in impaired ability to adapt to crisis situations [43]. Lower BDNF levels are associated with a decline in neuroprotection, and administration of BDNF has been shown to be protective in acute brain injury and infection models [44]–[45]. Maintenance of BDNF levels in the brain is also required for normal learning and memory function [46]. Additionally, BDNF levels have been linked to motor performance [47]. The lower levels of BDNF induced by malaria infection may have contributed to the observed deficits in learning, memory, and motor performance in mice treated with anti-malarials alone, while the quicker recovery and the higher levels achieved in rosiglitazone-treated mice may have conferred protection from neurological injury.

Here we report for the first time that circulating BDNF levels also decline in *P. falciparum* infection. Using samples collected during a randomized double-blind placebo-controlled trial of rosiglitazone adjunctive therapy in patients with uncomplicated *P. falciparum* infection, we observed a decrease in circulating BDNF in both placebo and rosiglitazone arms in the first 24 hours following admission. However, while BDNF levels remained low in the placebo group, the rosiglitazone treatment group had a significant increase in circulating BDNF levels. These findings imply that even uncomplicated falciparum malaria may be associated with some degree of neurological compromise or injury, at least as suggested by a persistent decrease in BDNF. This observation is in agreement with previous reports of impaired school performance in children with a history of uncomplicated malaria [4]. Our data also demonstrate that rosiglitazone adjunctive therapy may protect against neurological compromise in *P. falciparum* malaria by inducing established neuroprotective pathways such as BDNF.

It is important to note that our measurements of BDNF were limited to peripheral levels and may not necessarily reflect BDNF levels in the brain. However, previous studies in animal models have shown that circulating BDNF levels correlate with CNS levels of BDNF [48]. Thus circulating BDNF may be diagnostically useful as a biomarker for CNS injury in malaria infection, although this correlation will need to be confirmed in prospective studies.

Also of note is that the anti-malarial used in this trial was atovaquone-proguanil (which remains a 1^st^ line agent for the treatment of uncomplicated mefloquine-resistant malaria prevalent in the study site) rather than artemisinin-combination therapy (the current standard of care in Thailand). Although, the anti-malarial regimens differed between our animal model and our patient data, in both cases rosiglitazone was associated with induction of similar protective pathways indicating that its protective effects are independent of the anti-malarial used.

While PbA infection was not associated with a decline in NGF, we observed significant increases in NGF levels late in the course of treatment only in the mice that received rosiglitazone. NGF has been shown to promote neurogenesis and improve survival and differentiation of newly generated neurons in the adult brain, especially following injury [49]. Thus, the late increase in NGF levels in mice treated with rosiglitazone may have contributed to enhanced neurogenesis and recovery from CM-induced CNS injury.

Endothelial activation and loss of vascular integrity is central to the pathophysiology of CM, and vascular leakage has been shown to directly correlate with neurological disease onset in ECM [11]. Rosiglitazone adjunctive therapy contributed to the preservation of endothelial quiescence and vascular integrity in part by preventing malaria-induced alterations in the Ang-Tie2 axis. This was evidenced by the maintenance of peripheral Ang-1 levels in rosiglitazone-treated mice (Ang-1 sharply declined in infected mice), and a lower Ang-2 to Ang-1 mRNA ratio in the brain. A lower Ang-2 to Ang-1 ratio was also observed in patients with *P. falciparum* infection treated with rosiglitazone. Low Ang-1, elevated Ang-2, and an Ang-2 to Ang-1 ratio favouring Ang-2 have been reported in severe and cerebral malaria compared to uncomplicated malaria, and have been associated with mortality in CM [50]–[52].

Rosiglitazone adjunctive therapy was also associated with increased levels of the antioxidant enzymes SOD-1 and catalase in the brain, which correlated with protection from neurocognitive impairment. This is in agreement with previous observations showing that antioxidant adjunctive therapy was associated with reduced cognitive damage in mice [22]. Oxidative stress is thought to contribute to neuronal dysfunction in CM patients [10], and reduced anti-oxidant activity has been reported in CM patients and in ECM [23], [53]. Enhancement of anti-oxidant enzyme expression in endothelial and neuronal cells has been reported for PPARγ agonists, and is thought to contribute to the neuroprotective effects observed for PPARγ agonists in other models of CNS injury and disease [14].

Malaria immunopathogenesis is complex and therefore therapeutic interventions targeting a single pathway may not be sufficient to reduce mortality and morbidity in CM. We have demonstrated that rosiglitazone (which can exert its effects via activation of the nuclear transcription factor PPARγ and/or via PPARγ-independent mechanisms) can target multiple pathways implicated in the pathogenesis of CM including inflammation, oxidative stress, and endothelial activation, and is capable of initiating or enhancing neuroprotective mechanisms including the induction of neurotrophic factors. We provide direct evidence for the involvement of the Angiopoietin-Tie2 axis in mediating, at least in part, the observed neuroprotective effects of rosiglitazone. Future studies will be required to establish causality for the other implicated pathways, however, overall our data clearly demonstrate that rosiglitazone adjunctive therapy resulted in increased survival and protection from long-term neurocognitive impairment in ECM over what was observed with anti-malarial treatment alone. Importantly we provide evidence that rosiglitazone may also induce such putative protective mechanisms in falciparum-infected patients. Our findings are in agreement with the effects observed with PPARγ agonists in models of ischemic and hemorrhagic stroke, and CNS disease including Alzheimer's disease, multiple sclerosis, amyotrophic lateral sclerosis and Parkinson's disease [54]–[56]. PPARγ agonist use in these models and in patients was associated with reduced brain injury, attenuated neuronal loss, and improved neurological outcomes including motor and memory performance [57]–[61].

PPARγ agonists are currently approved for use in humans for the treatment of type II diabetes, which may accelerate their path to clinical evaluation and impact. Rare cardiac adverse events have been reported with long-term use of this class of drugs in elderly patients at high risk of cardiac disease; however in short-term use they have an excellent safety and tolerability profile. Rosiglitazone adjunctive therapy has already been found to be safe, well tolerated, and efficacious in adult patients with uncomplicated malaria [17], and short course therapy in a patient population that is typically at low risk of cardiac disease is unlikely to be associated with these types of adverse events.

In summary our results in an experimental model of CM and in patients with uncomplicated falciparum malaria suggest that PPARγ agonists are a promising adjunctive therapy for CM that may improve survival and prevent long-term neurocognitive impairment, and support the testing of PPARγ agonists in patients with CM.

## Materials and Methods

### Ethics statement

All animal experiments were approved by the University Health Network and the University of Toronto Animal Use Committees, and were performed according to the policies and guidelines of the Canadian Council on Animal Care.

Human plasma samples used in this study were collected from participants of a randomized double-blind placebo controlled trial testing the safety and efficacy of rosiglitazone adjunctive therapy in patients with *P. falciparum* malaria in Thailand performed during the period of December 2004 through December 2005 [17]. The study was approved by Mahidol University Research Ethics Committee, Bangkok, Thailand, and Toronto Academic Health Sciences Network Research, University Health Network. Written informed consent was obtained from all participating adult patients. For patients under the age of 18 written informed consent was obtained from their guardians. The trial was registered with ClinicalTrials.gov (identifier NCT00149383).

### Murine model of experimental cerebral malaria

*P. berghei* ANKA (PbA) (MR4, Bethesda MD) was maintained by passage in naive mice. Female 7–8 week old C57BL/6 mice (Charles River Laboratories) were infected with 1×10^6^ PbA parasitized erythrocytes by intraperitoneal (i.p.) injection (day 0 of infection) [16]. Mice were treated starting either on day 3 of infection, or on the onset of CM signs (day 5–6 of infection). For the day 3 experiments (Figure S1A), mice were treated with 10 mg/kg artesunate suspended in PBS i.p. on day 3, 4, 5 and 6 post-infection, and either 2.5 mg/kg rosiglitazone in 100 µl of saline (rosiglitazone group), or 100 µl of saline alone (control group) once daily by oral gavage starting on day 3 until the end of the experiment. Otherwise, mice were treated with 10 mg/kg artesunate i.p. for two days starting on the onset of CM signs, and either 2.5 mg/kg rosiglitazone or saline as a control by oral gavage daily starting at the same time as artesunate treatment and lasting until the end of the experiment (Figure S1B). Parasitemia was monitored daily by Giemsa-stained thin-blood smears.

For all drug-cure experiments (for behavioural and MRI testing) mice were treated at the onset of CM signs with 7 once daily doses of 100 mg/kg artesunate, 2 doses of 15 mg/kg mefloquine on the first and last day of treatment, and 7 once daily doses of 2.5 mg/kg rosiglitazone (Figure S1C). Parasitemia was monitored weekly. Mice remained parasite-free for the duration of behavioural testing.

Ang-1^del^ mice on a BALB/c background were the kind gift of Dr. Susan Quaggin (Mount Sinai Hospital, Toronto, Canada) [20]. BALB/c wild-type breeding pairs were purchased from the Jackson Laboratory and bred in house. Cerebral malaria score for each mouse was assessed on multiple days by a single observer, who was blinded to genotype and treatment allocation, using a modified version of the rapid coma and behavioural score [21].

### Analysis of systemic endothelial activation

Heparinized plasma was collected via saphenous vein or by cardiac puncture and frozen at −80°C. Plasma levels of Ang-1 and sICAM-1 were determined by ELISA (R&D Systems, Minneapolis, MN).

### Analysis of vascular permeability

On day 10 post-infection drug-treated mice were injected i.p. with 300 µl of 2% Evans blue. 2 h post-injection mice were euthanized using isoflurane, and perfused with 20 mls of PBS. Brains were collected, photographed, and placed in formamide for 48 h to extract the Evans blue. Evans blue was quantified using a spectrophotometer at 605 nm and compared to a standard curve.

### Luciferase imaging of parasite distribution

Mice were infected i.p. with 1×10^6^ PEs of a luciferase-expressing PbA (PbGFP-LUC(con)) and treated as described above [19]. Imaging was conducted on day 10 post-infection as previously described [62].

### Histological analysis

Brains were collected on day 10 post-infection from parasitemia-matched mice, following perfusion with 20 mls of PBS, were fixed in 4% formalin for 3 days, and then paraffin embedded. 4 µm sagittal slices were stained with H&E and examined blinded to the treatment group, to quantify the number of hemorrhages. The entire brain section was scanned and all hemorrhages (defined as collections of RBC observed outside of blood vessels) were recorded.

### Brain quantitative real time PCR

Total RNA was isolated from snap frozen brain tissue after homogenization in TRIzol (1 mL/100 mg tissue; Invitrogen, Burlington, ON) according to manufacturer's protocol. Extracted RNA (1μg/sample) was treated with DNase I (Fermentas, Burlington, ON), and reverse transcribed to cDNA (BioRad, Mississauga, ON). cDNA was amplified in triplicate with SYBR Green master mix (Roche, Laval, QC) in the presence of 1μM of forward and reverse primers in a Light Cycler 480 (Roche, Laval, QC). Relative amounts of transcript were calculated by the comparative Ct method (2^−ΔΔCT^). GAPDH was used as an endogenous control. The primer sequences are shown in Table S1.

### Behavioural testing

Behavioural testing began 2 months following completion of treatment, when the mice were approximately 4 months of age, and lasted approximately 3 weeks. During testing, the experimenter alternated between mice from each experimental group. Tests were performed in the following order: open field test (OFT), novel object recognition test (NOR), tail suspension test (TST), rotarod test, and contextual fear conditioning test (CFC). Methodological details for the OFT, TST, and CFC are included in the Text S1. Supplemental Methods.

For the NOR test, mice were habituated to the testing apparatus (empty clear plastic mouse cages) for 10 minutes over 6 daily sessions. On the test day, each animal was exposed for 10 minutes to a LEGO construct (LEGO Group, Billund, Denmark) and a Hot Wheels car (Mattel, Inc., El Segundo, CA, USA). The objects were previously determined to be of matched saliency in mice. All tests were video recorded suing ANYMAZE software. Time spent exploring both objects was recorded. Exploration was coded when the mouse touched an object with its forepaws or snout, bit, licked, or sniffed the objects from a distance of no more than 1.5 cm. Following exploration mice were returned to their home cage. Three hours after the initial exposure, mice were returned to the test cage and exposed for 5 minutes to one object from the original test pair and one novel object. The possible confound of mice exhibiting preference for the right or left side of the cage was addressed by counterbalancing for placements of the new object. A “preference index” (PI) was calculated as: PI  =  tn/(tn + tf), wherein “tn” represents time exploring a novel object or object in a novel placement area and “tf” the duration of familiar object exploration [63]. All animals with a total exploration time of less than 10 seconds in either the exploration or testing phase were removed from the analysis.

For the rotarod test, an Economex accelerating rotarod (Columbus Instruments, Columbus, OH, USA) was used to assess postural sensorimotor impairment. Mice were placed on the rod as it turned at a constant speed of 4 rpm. The latency to fall was recorded in five daily trials conducted at 30 min intervals, performed for 3 consecutive days. The daily performance score for each animal was the sum latency to fall off the apparatus across all five daily trials. To measure adaptive performance on the 4^th^ and 5^th^ day mice were placed on the rod starting at a constant speed of 4 rpm, with an acceleration of 0.2 rpm/s for 5 successive trials over a 30 min interval. Latency to fall was recorded for each trial. A daily performance score for each animal was obtained by summing latency over the 5 trials.

### Ex vivo Magnetic Resonance Imaging

Specimen preparation for MRI was as described in [64]. Upon completion of all behavioural testing animals were anesthetized with a ketamine (150 mg/kg)/xylazine (10 mg/kg) mix and perfused transcardially with 30 mL of solution A (1×PBS +2mM Prohance (a contrast agent used for MRI) +1 µL/mL Heparin) and then with 30 mL of solution B (1×PBS +4% Paraformaldehyde +2 mM Prohance). Animals were then decapitated and skin, cartilage and lower jaw was removed. Tissue was left at 4°C for 24 h in 10 mL of solution B and then transferred into 10 mL of solution C (1×PBS +0.02% sodium azide +2 mM Prohance) for storage prior to scanning. Tissue was left in solution C for a maximum of 6 months prior to scanning. The MRI methods used here have previously been described in detail [28], [31]. An anatomical mouse brain atlas was used to define brain regions and structures and to compute volumes for each mouse brain [31]–[32]. Additional details can be found in the Text S1. Supplemental Methods.

### Randomized clinical trial patient population and analysis of biomarkers

Ang-1, Ang-2, and BDNF levels were assessed by ELISA (R&D Systems, Minneapolis, MN) in plasma collected from participants of a randomized double-blind placebo controlled trial testing the safety and efficacy of rosiglitazone adjunctive therapy in patients with *P. falciparum* malaria in Thailand. The details of this trial are described in [17].

### Statistical analysis

Survival studies were performed at least 3 times. Other experiments were repeated at least once. Statistical significance for survival studies was assessed by log-rank test. Other comparisons were assessed by Mann-Whitney test or Students t-test, or one-way or two-way ANOVA with post-hoc tests. Statistical analyses were performed using GraphPad Prism software (LaJolla, CA). Linear regression modeling was used to identify brain regions that differed significantly between treatment groups.

## Supporting Information

Figure S1 **Treatment protocols.** (A) Treatment protocol commencing on day 3 post-infection. (B) Treatment protocol commencing at the onset of CM signs, between day 5 and 6. (C) Treatment protocol for drug-cure experiments. All mice were infected with 1 million *P. berghei* ANKA parasitized erythrocytes by i.p. injection on day 0. Artesunate and mefloquine were administered by i.p. injection. Mice received either rosiglitazone or saline as a control. Both were administered by gavage.
(TIF)

Figure S2 **Circulating levels of angiopoietin-1 in mice infected with** ***P. berghei*** **ANKA are increased by rosiglitazone adjunctive therapy.** Mice infected with *P. berghei* ANKA were left untreated (dashed black), or were treated with either rosiglitazone alone starting either pre-infection (solid blue) or on day 3 post-infection (dashed blue), or with artesunate plus saline (solid black) or artesunate plus rosiglitazone (solid red) starting at day 3 post-infection. Survival curves are shown in (A), N = 10/group. Statistical comparisons by Logrank test, p = 0.0042 for artesunate vs. artesunate + rosiglitazone; p = 0.037 for untreated vs. rosiglitazone (pre-infection); p<0.0001 for untreated vs. artesunate or vs. artesunate + rosiglitazone. Serial plasma samples were collected from each group on day 0, 5, 7, and 13 of infection and angiopoietin-1 levels were analysed by ELISA. Ang-1 levels normalised to d0 values are shown in (B). Data were analysed by two-way ANOVA with Bonferroni post-test. N = 6 per group, however for the untreated and rosiglitazone-only treated groups N = 1 or 2 for day 7 since mice had began succumbing to their infection by this time point. By day 13 all mice had succumbed to their infection in the untreated and rosiglitazone-only treated groups. P<0.01 on day 5 for artesunate vs. art + rosiglitazone, and for artesunate vs. rosiglitazone (pre-infection).
(TIF)

Figure S3 **Brain expression levels of angiopoietin-1 are increased, and the ang-2 to ang-1 ratio is decreased in mice infected with** ***P. berghei*** **ANKA treated with rosiglitazone adjunctive therapy.** Mice infected with *P. berghei* ANKA were treated with artesunate plus saline (grey bars), or artesunate plus rosiglitazone (red bars) starting at the onset of CM signs. Expression of ang-1 (A) and ang-2 (B) mRNA was assessed in brain homogenates collected from uninfected mice (day 0), infected mice prior to the initiation of therapy (day 5), and infected mice following treatment initiation (on day 7 and 9 post-infection). The ratio of ang-2:ang-1 was also calculated (C). Data were analysed by Kruskal-Wallis test with Dunn's post-test, N = 6/group. Significant differences were observed for ang-1 and the ang2:ang1 ratio on day 7. Ang-2 levels were lower in the rosiglitazone-treated group but did not reach significance. * p<0.05.
(TIF)

Figure S4 **Rosiglitazone improves survival and reduces disease severity in mice sufficient in angiopoietin-1 but not in mice with an angiopoietin-1 deletion.** Mice either sufficient for Ang-1 (solid lines) (A–B) or that have one Ang-1 allele deleted (Ang-1^del^; dashed lines) (C–D) were infected with *P. berghei* ANKA and treated with artesunate plus saline (Art; shown in black) or artesunate plus rosiglitazone (Art + Rosi; shown in red) starting on day 5.5 post-infection. Survival curves are shown in (A) and (C). Rosiglitazone adjunctive therapy improved survival only in the Ang-1 sufficient mice (P<0.05, by Logrank test, N = 11–15/group), but not in the Ang-1^del^ mice. A modified coma and behavioural score was used to assess disease severity [1]. Mice were assessed on consecutive days as shown. Data for the Ang-1 sufficient mice are shown in (B) and for the Ang-1^del^ in (D). Rosiglitazone adjunctive therapy reduced disease severity (i.e. higher score) only in the Ang-1 sufficient mice. Data are means with SEM, N = 11–15/group. Statistical comparison by two-way ANOVA with Bonferroni post-test. * P<0.05, ** P<0.01. (E) Baseline plasma levels of angiopoietin-1 in Ang-1 sufficient and Ang-1^del^ mice. P<0.0001 by Mann Whitney test.
(TIF)

Figure S5 **HO-1 expression levels in the brain of mice infected with** ***P. berghei*** **ANKA are unaffected by rosiglitazone adjunctive therapy.** Mice infected with *P. berghei* ANKA were treated with artesunate plus saline (grey bars), or artesunate plus rosiglitazone (red bars) starting at the onset of CM signs. Expression of HO-1 mRNA was assessed in brain homogenates collected from uninfected mice (day 0), infected mice prior to the initiation of therapy (day 5), and infected mice following treatment initiation (on day 7 and 9 post-infection). Data were analysed by Kruskal-Wallis test with Dunn's post-test, N = 6 per group.
(TIF)

Figure S6 **Mobility and exploration time in the novel object recognition test are similar between treatment groups.** Mice infected with *P. berghei* ANKA were drug-cured at the onset of CM with either artesunate/mefloquine plus saline (grey bars), or artesunate/mefloquine plus rosiglitazone (red bars). Drug-treated uninfected mice were used as controls (white bars). Testing was performed 2 months following completion of treatment. Data for mobility in the initial exploration round of the NOR test are shown in (A). Data on the mobility of the mice during testing are shown in (B). Data on the total initial exploration time are shown in (C), and for the total exploration time during testing in (D). All data are means with standard deviations. No significant differences are seen between treatment groups, as assessed by one-way ANOVA. Abbreviations: Art, artesunate; M, mefloquine; Rosi, rosiglitazone.
(TIF)

Figure S7 **Performance in the Open Field, Tail Suspension, and Contextual Fear Conditioning Test do not differ between treatment groups.** Mice infected with *P. berghei* ANKA were drug-cured at the onset of CM with either artesunate/mefloquine plus saline (grey bars) or artesunate/mefloquine plus rosiglitazone (red bars). Drug-treated uninfected mice were used as controls (white bars). Testing was performed 2 months following completion of treatment. Data from the Open Field test are shown in (A). Total distance covered during the Open Field Test is shown. Data from the Tail Suspension test are shown in (B). Data from the Contextual Fear Conditioning test are shown in (C). Freezing assessed prior to the conditioning stimulus and post the conditioning stimulus is shown. All data shown are medians with range. No significant differences were observed between groups as assessed by Kruskal-Wallis test.
(TIF)

Figure S8 **Rosiglitazone adjunctive therapy protects mice from malaria-induced brain atrophy.** Following completion of all behavioural testing mice were sacrificed and their brains scanned using magnetic resonance imaging (MRI). This was followed by image registration and volumetric analysis of the brain volume of 62 distinct regions. Linear regression analysis was performed to identify areas that differed significantly between the mice treated with artesunate/mefloquine plus saline (grey bars), and the mice treated with artesunate/mefloquine plus rosiglitazone (red bars). Significant differences were observed in (A) the cerebral penduncle, (B) the fimbria, (C) the posterior commissure, (D) the colliculus inferior, (E) the internal capsule, and (F) the stria terminalis. N = 13 for artesunate/mefloquine, N = 10 for artesunate/mefloquine + rosiglitazone. The median volume for uninfected mice is shown as a dashed line (N = 10 for uninfected control).
(TIF)

Table S1 **Primers used for qRT-PCR analysis of brain homogenates.**
(DOC)

Text S1 **Supplemental methods.**
(DOC)

## References

[1] Dondorp A, Nosten F, Stepniewska K, Day N, White N, et al (2005) Artesunate versus quinine for treatment of severe falciparum malaria, a randomised trial. Lancet 366: 717–725. 16125588 10.1016/S0140-6736(05)67176-0

[2] Dondorp AM, Fanello CI, Hendriksen IC, Gomes E, Seni A, et al (2010) Artesunate versus quinine in the treatment of severe falciparum malaria in African children (AQUAMAT), an open-label, randomised trial. Lancet 376: 1647–1657. 21062666 10.1016/S0140-6736(10)61924-1 PMC3033534

[3] John CC, Kutamba E, Mugarura K, Opoka RO (2010) Adjunctive therapy for cerebral malaria and other severe forms of Plasmodium falciparum malaria. Expert Rev Anti Infect Ther 8: 997–1008. 20818944 10.1586/eri.10.90 PMC2987235

[4] John CC, Bangirana P, Byarugaba J, Opoka RO, Idro R, et al (2008) Cerebral malaria in children is associated with long-term cognitive impairment. Pediatrics 122: e92–99. 18541616 10.1542/peds.2007-3709 PMC2607241

[5] Boivin MJ, Bangirana P, Byarugaba J, Opoka RO, Idro R, et al (2007) Cognitive impairment after cerebral malaria in children, a prospective study. Pediatrics 119: e360–366. 17224457 10.1542/peds.2006-2027 PMC2743741

[6] Birbeck G, Molyneux ME, Kaplan PW, Seydel KB, Chimalizeni YF, et al (2010) Blantyre Malaria Project Epilepsy Study (BMPES) of neurological outcomes in retinopathy-positive paediatric cerebral malaria survivors: a prospective cohort study. Lancet Neurol 9: 1173–1181. 21056005 10.1016/S1474-4422(10)70270-2 PMC2988225

[7] Fernando SD, Rodrigo C, Rajapakse S (2010) The hidden burden of malaria, cognitive impairment following infection. Malaria J 9: 366. 10.1186/1475-2875-9-366 PMC3018393 21171998

[8] Taylor TE, Fu WJ, Carr RA, Whitten RO, Mueller JS, et al (2004) Differentiating the pathologies of cerebral malaria by postmortem parasite counts. Nat Med 10: 143–145. 14745442 10.1038/nm986

[9] White VA, Lewallen S, Beare N, Kayira K, Carr RA, et al (2001) Correlation of retinal haemorrhages with brain haemorrhages in children dying of cerebral malaria in Malawi. Trans R Soc Trop Med Hyg 95: 618–621. 11816433 10.1016/s0035-9203(01)90097-5

[10] Medana IM, Day NP, Hien TT, Mai NT, Bethell D, et al (2002) Axonal injury in cerebral malaria. Am J Pathol 160: 655–666. 11839586 10.1016/S0002-9440(10)64885-7 PMC1850649

[11] Nacer A, Movilla A, Baer K, Mikolajczak SA, Kappe SH, et al (2012) Neuroimmunological blood brain opening in experimental cerebral malaria. PloS Pathog 8: e1002982. 23133375 10.1371/journal.ppat.1002982 PMC3486917

[12] Berger J, Moller DE (2002) The mechanism of action of PPARs. Ann Rev Med 53: 409–435. 11818483 10.1146/annurev.med.53.082901.104018

[13] Lehrke M, Lazar MA (2005) The many faces of PPARgamma. Cell 123: 993–999. 16360030 10.1016/j.cell.2005.11.026

[14] Kapadia R, Yi JH, Vemuganti R (2008) Mechanisms of anti-inflammatory and neuroprotective actions of PPAR-gamma agonists. Front Biosci 13: 1813–1826. 17981670 10.2741/2802 PMC2734868

[15] Jin J, Albertz J, Guo Z, Peng Q, Rudow G, et al (2013) Neuroprotective effects of PPAR-γ agonist rosiglitazone in N171-82Q mouse model of Huntington's disease. J Neurochem 125: 410–419. 23373812 10.1111/jnc.12190 PMC3642978

[16] Serghides L, Patel SN, Ayi K, Lu Z, Gowda DC, et al (2009) Rosiglitazone modulates the innate immune response to Plasmodium falciparum infection and improves outcome in experimental cerebral malaria. J Infect Dis 199: 1536–1545. 19392627 10.1086/598222 PMC2854576

[17] Boggild AK, Krudsood S, Patel SN, Serghides L, Tangpukdee N, et al (2009) Use of peroxisome proliferator-activated receptor gamma agonists as adjunctive treatment for Plasmodium falciparum malaria, a randomized, double-blind, placebo-controlled trial. Clin Infect Dis 49: 841–849. 19673614 10.1086/605431

[18] Kim H, Higgins S, Liles WC, Kain KC (2011) Endothelial activation and dysregulation in malaria, a potential target for novel therapeutics. Curr Opion Hematol 18: 177–185. 10.1097/MOH.0b013e328345a4cf 21423010

[19] Franke-Fayard B, Waters AP, Janse CJ (2006) Real-time in vivo imaging of transgenic bioluminescent blood stages of rodent malaria parasites in mice. Nat Protoc 1: 476–485. 17406270 10.1038/nprot.2006.69

[20] Jeansson M, Gawlik A, Anderson G, Li C, Kerjaschki D, et al (2011) Angiopoietin-1 is essential in mouse vasculature during development and in response to injury. J Clin Invest 121: 2278–2289. 21606590 10.1172/JCI46322 PMC3104773

[21] Carroll RW, Wainwright MS, Kim KY, Kidambi T, Gomez ND, et al (2010) A rapid murine coma and behavior scale for quantitative assessment of murine cerebral malaria. PLoS One 5: e13124. 20957049 10.1371/journal.pone.0013124 PMC2948515

[22] Reis PA, Comim CM, Hermani F, Silva B, Barichello T, et al (2010) Cognitive dysfunction is sustained after rescue therapy in experimental cerebral malaria, and is reduced by additive antioxidant therapy. PLoS Pathog 24: e1000963. 10.1371/journal.ppat.1000963 PMC2891838 20585569

[23] Pabon A, Carmona J, Burgos LC, Blair S (2003) Oxidative stress in patients with non-complicated malaria. Clin Biochem 36: 71–78. 12554064 10.1016/s0009-9120(02)00423-x

[24] Skaper SD (2012) The neurotrophin family of neurotrophic factors: an overview. Methods Mol Biol 846: 1–12. 22367796 10.1007/978-1-61779-536-7_1

[25] Linares M, Marin-Garcia P, Perez-Benavente S, Sanchez-Nogueiro J, Puyet A, et al (2013) Brain-derived neurotrophic factor and the course of experimental cerebral malaria. Brain Res 1490: 210–224. 23123703 10.1016/j.brainres.2012.10.040

[26] Huang EJ, Reichardt LF (2003) Trk receptors, roles in neuronal signal transduction. Annu Rev Biochem 72, 609–642. 10.1146/annurev.biochem.72.121801.161629 12676795

[27] Dai M, Reznik SE, Spray DC, Weiss LM, Tanowitz HB, et al (2010) Persistent cognitive and motor deficits after successful antimalarial treatment in murine cerebral malaria. Microbes Infect 12: 1198–1207. 20800692 10.1016/j.micinf.2010.08.006 PMC3048460

[28] Lerch JP, Yiu AP, Martinez-Canabal A, Pekar T, Bohbot VD, et al (2011) Maze training in mice induces MRI-detectable brain shape changes specific to the type of learning. Neuroimage 54: 2086–2095. 20932918 10.1016/j.neuroimage.2010.09.086

[29] Potchen MJ, Birbeck GL, Demarco JK, Kampondeni SD, Beare N, et al (2010) Neuroimaging findings in children with retinopathy-confirmed cerebral malaria. Eur J Radiol 74: 262–268. 19345538 10.1016/j.ejrad.2009.02.010 PMC3786197

[30] Kampondeni SD, Potchen MJ, Beare NA, Seydel KB, Glover SJ, et al (2013) MRI findings in a cohort of brain injured survivors of pediatric cerebral malaria. Am J Trop Med Hyg 88: 542–546. 23339204 10.4269/ajtmh.12-0538 PMC3592538

[31] Lerch JP, Sled JG, Henkelman RM (2011) MRI phenotyping of genetically altered mice. Methods Mol Biol 711: 349–361. 21279611 10.1007/978-1-61737-992-5_17

[32] Dorr AE, Lerch JP, Spring S, Kabani N, Henkelman RM (2008) High resolution three-dimensional brain atlas using an average magnetic resonance image of 40 adult C57Bl/6J mice. Neuroimage 42: 60–69. 18502665 10.1016/j.neuroimage.2008.03.037

[33] Boivin MJ, Gladstone MJ, Vokhiwa M, Birbeck GL, Magen JG, et al (2011) Developmental outcomes in Malawian children with retinopathy-positive cerebral malaria. Trop Med Int Health 16: 263–271. 21143354 10.1111/j.1365-3156.2010.02704.x PMC3213405

[34] Kihara M, Carter JA, Holding PA, Vargha-Khadem F, Scott RC, et al (2009) Impaired everyday memory associated with encephalopathy of severe malaria, the role of seizures and hippocampal damage. Malaria J 8: 273. 10.1186/1475-2875-8-273 PMC2794875 19951424

[35] Carter JA, Mung'ala-Odera V, Neville BG, Murira B, Mturi N, et al (2005) Persistent neurocognitive impairments associated with severe falciparum malaria in Kenyan children. J Neurol Neurosurg Psychiatry 76: 476–481. 15774431 10.1136/jnnp.2004.043893 PMC1739592

[36] Masi G, Brovedani P (2011) The hippocampus, neurotrophic factors and depression, possible implications for the pharmacotherapy of depression. CNS Drugs 25: 913–931. 22054117 10.2165/11595900-000000000-00000

[37] Van der Werf YD, Witter MP, Uylings HB, Jolles J (2000) Neuropsychology of infarctions in the thalamus, a review. Neuropsychologia 38, 613–627. 10.1016/s0028-3932(99)00104-9 10689038

[38] Pozzilli C, Bastianello S, Padovani A, Passafiume D, Millefiorini E, et al (1991) Anterior corpus callosum atrophy and verbal fluency in multiple sclerosis. Cortex 27: 441–445. 1743039 10.1016/s0010-9452(13)80039-1

[39] Hines M, Chiu L, McAdams LA, Bentler PM, Lipcamon J (1992) Cognition and the corpus callosum, verbal fluency, visuospatial ability, and language lateralization related to midsagittal surface areas of callosal subregions. Behav Neurosci 106: 3–14. 1554435 10.1037//0735-7044.106.1.3

[40] Aggleton JP, O'Mara SM, Vann SD, Wright NF, Tsanov M, et al (2010) Hippocampal-anterior thalamic pathways for memory, uncovering a network of direct and indirect actions. Eur J Neurosci 31: 2292–2307. 20550571 10.1111/j.1460-9568.2010.07251.x PMC2936113

[41] Potchen MJ, Kampondeni SD, Seydel KB, Birbeck GL, Hammond CA, et al (2012) Acute brain MRI findings in 120 Malawian children with cerebral malaria, new insights into an ancient disease. AJNR Am J Neuroradiol 33: 1740–1746. 22517285 10.3174/ajnr.A3035 PMC3779545

[42] Comim CM, Reis PA, Frutuso VS, Fries GR, Fraga DB, et al (2012) Effects of experimental cerebral malaria in memory, brain-derived neurotrophic factor and acetylcholinesterase activity in the hippocampus of survivor mice. Neurosci Lett 523: 104–107. 22750161 10.1016/j.neulet.2012.06.051

[43] Gomez-Palacio-Schjetnan A, Escobar ML (2013) Neurotrophins and synaptic plasticity. Curr Top Behav Neurosci 15: 117–136. 23519767 10.1007/7854_2012_231

[44] Shi Q, Zhang P, Zhang J, Chen X, Lu H, et al (2009) Adenovirus-mediated brain-derived neurotrophic factor expression regulated by hypoxia response element protects brain from injury of transient middle cerebral artery occlusion in mice. Neurosci Lett 465: 220–225. 19703519 10.1016/j.neulet.2009.08.049

[45] Barichello T, Belarmino E Jr, Comim CM, Cipriano AL, Generoso JS, et al (2010) Correlation between behavioral deficits and decreased brain-derived neurotrophic factor in neonatal meningitis. J Neuroimmunol 223: 73–76. 20452683 10.1016/j.jneuroim.2010.04.004

[46] Bekinschtein P, Cammarota M, Katche C, Slipczuk L, Rossato JI, et al (2008) BDNF is essential to promote persistence of long-term memory storage. Proc. Natl Acad Sci USA 105: 2711–2716. 10.1073/pnas.0711863105 PMC2268201 18263738

[47] Canals JM, Pineda JR, Torres-Peraza JF, Bosch M, Martin-Ibanez R, et al (2004) Brain-derived neurotrophic factor regulates the onset and severity of motor dysfunction associated with enkephalinergic neuronal degeneration in Huntington's disease. J Neurosci 24: 7727–7739. 15342740 10.1523/JNEUROSCI.1197-04.2004 PMC6729627

[48] Klein AB, Williamson R, Santini MA, Clemmensen C, Ettrup A, et al (2011) Blood BDNF concentrations reflect brain-tissue BDNF levels across species. Int J Neuropsychopharmacol 14: 347–353. 20604989 10.1017/S1461145710000738

[49] Sofroniew MV, Howe CL, Mobley WC (2001) Nerve growth factor signaling, neuroprotection, and neural repair. Annu Rev Neurosci 24: 1217–1281. 11520933 10.1146/annurev.neuro.24.1.1217

[50] Lovegrove FE, Tangpukdee N, Opoka RO, Lafferty EI, Rajwans N, et al (2009) Serum angiopoietin-1 and -2 levels discriminate cerebral malaria from uncomplicated malaria and predict clinical outcome in African children. PLoS One 4: e4912. 19300530 10.1371/journal.pone.0004912 PMC2657207

[51] Conroy AL, Glover SJ, Hawkes M, Erdman LK, Seydel KB, et al (2012) Angiopoietin-2 levels are associated with retinopathy and predict mortality in Malawian children with cerebral malaria, a retrospective case-control study. Crit Care Med 40: 952–959. 22343839 10.1097/CCM.0b013e3182373157 PMC3284252

[52] Yeo TW, Lampah DA, Gitawati R, Tjitra E, Kenangalem E, et al (2008) Angiopoietin-2 is associated with decreased endothelial nitric oxide and poor clinical outcome in severe falciparum malaria. Proc Natl Acad Sci U S A 105: 17097–17102. 18957536 10.1073/pnas.0805782105 PMC2575222

[53] Narsaria N, Mohanty C, Das BK, Mishra SP, Prasad R (2012) Oxidative stress in children with severe malaria. J Trop Pediatr 58: 147–150. 21602230 10.1093/tropej/fmr043

[54] Drew PD, Xu J, Racke MK (2008) PPAR-gamma, therapeutic potential for multiple sclerosis. PPAR Res 2008: 627463. 18604287 10.1155/2008/627463 PMC2441778

[55] Vemuganti R (2008) Therapeutic potential of PPARγ activation in stroke. PPAR Res 2008: 461981. 21909480 10.1155/2008/461981 PMC2293414

[56] Bright JJ, Kanakasabai S, Chearwae W, Chakraborty S (2008) PPAR regulation of inflammation signaling in CNS diseases. PPAR Res 2008: 658520. 18670616 10.1155/2008/658520 PMC2490815

[57] Mandrekar-Colucci S, Karlo JC, Landreth GE (2012) Mechanisms underlying the rapid peroxisome proliferator-activated receptor-γ-mediated amyloid clearance and reversal of cognitive deficits in a murine model of Alzheimer's disease. J Neurosci 32: 10117–10128. 22836247 10.1523/JNEUROSCI.5268-11.2012 PMC3433721

[58] Escribano L, Simón AM, Pérez-Mediavilla A, Salazar-Colocho P, Río JD, et al (2009) Rosiglitazone reverses memory decline and hippocampal glucocorticoid receptor down-regulation in an Alzheimer's disease mouse model. Biochem Biophys Res Commun 379: 406–410. 19109927 10.1016/j.bbrc.2008.12.071

[59] Schintu N, Frau L, Ibba M, Caboni P, Garau A, et al (2009) PPAR-gamma-mediated neuroprotection in a chronic mouse model of Parkinson's disease. Eur J Neurosci 29: 954–963. 19245367 10.1111/j.1460-9568.2009.06657.x

[60] Watson GS, Cholerton BA, Reger MA, Baker LD, Plymate SR, et al (2005) Preserved cognition in patients with early Alzheimer disease and amnestic mild cognitive impairment during treatment with rosiglitazone, a preliminary study. Am J Geriatr Psychiatry 13: 950–958. 16286438 10.1176/appi.ajgp.13.11.950

[61] Kaiser CC, Shukla DK, Stebbins GT, Skias DD, Jeffery DR, et al (2009) A pilot test of pioglitazone as an add-on in patients with relapsing remitting multiple sclerosis. J Neuroimmunol 211: 124–130. 19446890 10.1016/j.jneuroim.2009.04.011

[62] Serghides L, Kim H, Lu Z, Kain DC, Miller C, et al (2011) Inhaled nitric oxide reduces endothelial activation and parasite accumulation in the brain, and enhances survival in experimental cerebral malaria. PLoS One 6: e27714. 22110737 10.1371/journal.pone.0027714 PMC3218025

[63] Francis BM, Kim J, Barakat ME, Fraenki S, Yucel YH, et al (2012) Object recognition memory and BDNF expression are reduced in young TgCRND8 mice. Neurobiol Aging 33: 555–563. 20447730 10.1016/j.neurobiolaging.2010.04.003 PMC3411544

[64] Cahill LS, Laliberte CL, Ellegood J, Spring S, Gleave JA, et al (2012) Preparation of fixed mouse brains for MRI. Neuroimage 60: 933–939. 22305951 10.1016/j.neuroimage.2012.01.100

