# Supplementary figures and images for: PPARγ Agonists Improve Survival and Neurocognitive Outcomes in Experimental Cerebral Malaria and Induce Neuroprotective Pathways in Human Malaria
Source: PLoS Pathog. 2014 Mar 6;10(3):e1003980. doi: 10.1371/journal.ppat.1003980 (PMC3946361; doi:10.1371/journal.ppat.1003980)

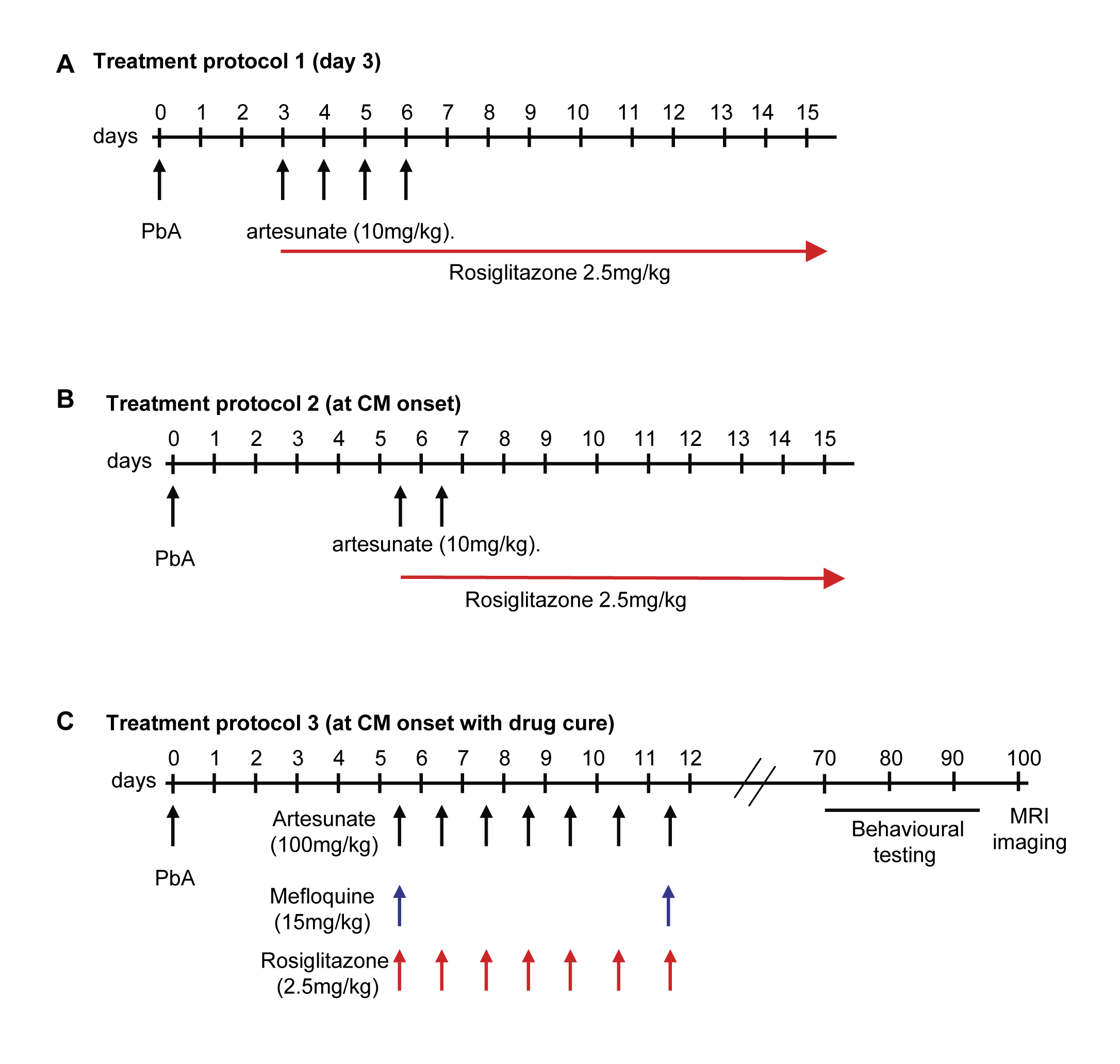

Supplement: Figure S1 — Treatment protocols. (A) Treatment protocol commencing on day 3 post-infection. (B) Treatment protocol commencing at the onset of CM signs, between day 5 and 6. (C) Treatment protocol for drug-cure experiments. All mice were infected with 1 million P. berghei ANKA parasitized erythrocytes by i.p. injection on day 0. Artesunate and mefloquine were administered by i.p. injection. Mice received either rosiglitazone or saline as a control. Both were administered by gavage. (TIF) [file ppat.1003980.s001.tif]

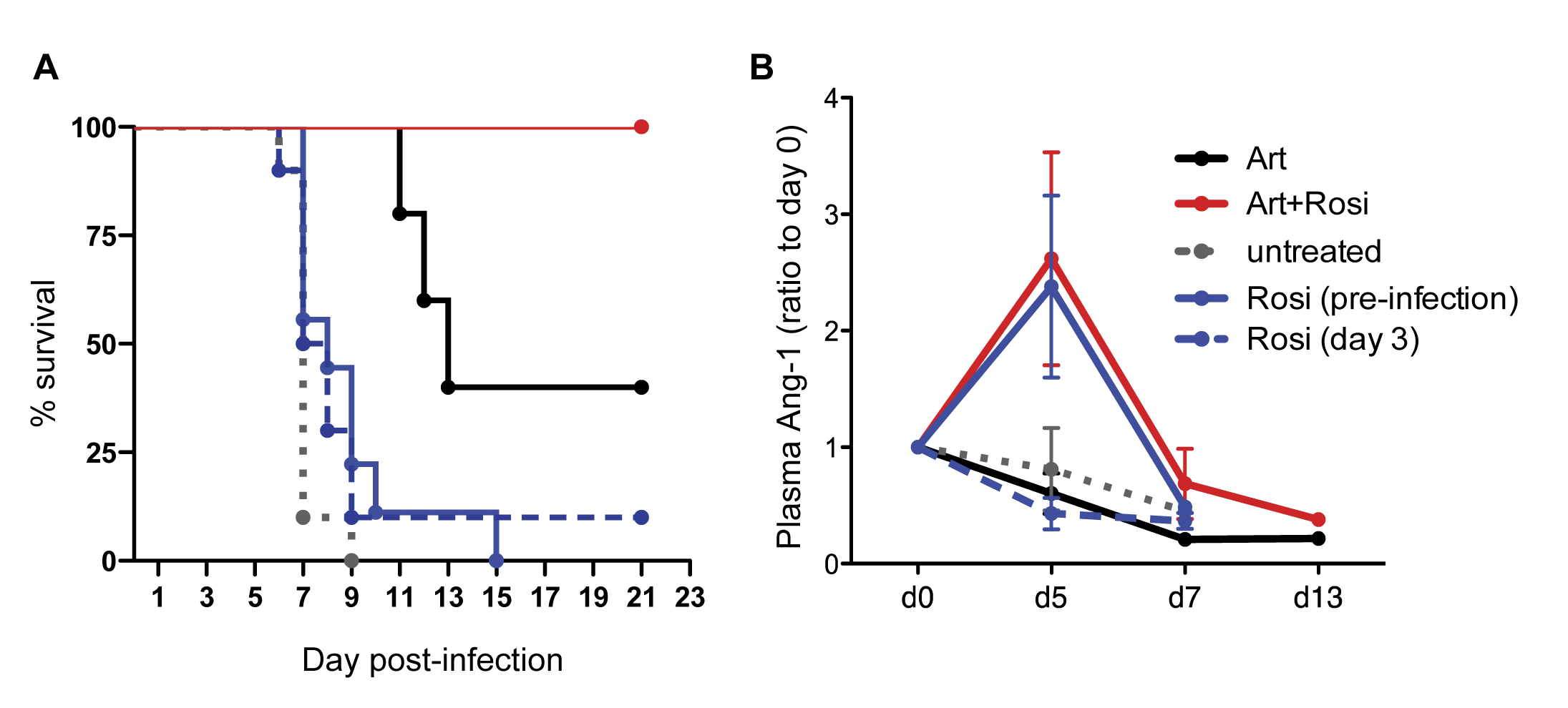

Supplement: Figure S2 — Circulating levels of angiopoietin-1 in mice infected with P. berghei ANKA are increased by rosiglitazone adjunctive therapy. Mice infected with P. berghei ANKA were left untreated (dashed black), or were treated with either rosiglitazone alone starting either pre-infection (solid blue) or on day 3 post-infection (dashed blue), or with artesunate plus saline (solid black) or artesunate plus rosiglitazone (solid red) starting at day 3 post-infection. Survival curves are shown in (A), N = 10/group. Statistical comparisons by Logrank test, p = 0.0042 for artesunate vs. artesunate + rosiglitazone; p = 0.037 for untreated vs. rosiglitazone (pre-infection); p<0.0001 for untreated vs. artesunate or vs. artesunate + rosiglitazone. Serial plasma samples were collected from each group on day 0, 5, 7, and 13 of infection and angiopoietin-1 levels were analysed by ELISA. Ang-1 levels normalised to d0 values are shown in (B). Data were analysed by two-way ANOVA with Bonferroni post-test. N = 6 per group, however for the untreated and rosiglitazone-only treated groups N = 1 or 2 for day 7 since mice had began succumbing to their infection by this time point. By day 13 all mice had succumbed to their infection in the untreated and rosiglitazone-only treated groups. P<0.01 on day 5 for artesunate vs. art + rosiglitazone, and for artesunate vs. rosiglitazone (pre-infection). (TIF) [file ppat.1003980.s002.tif]

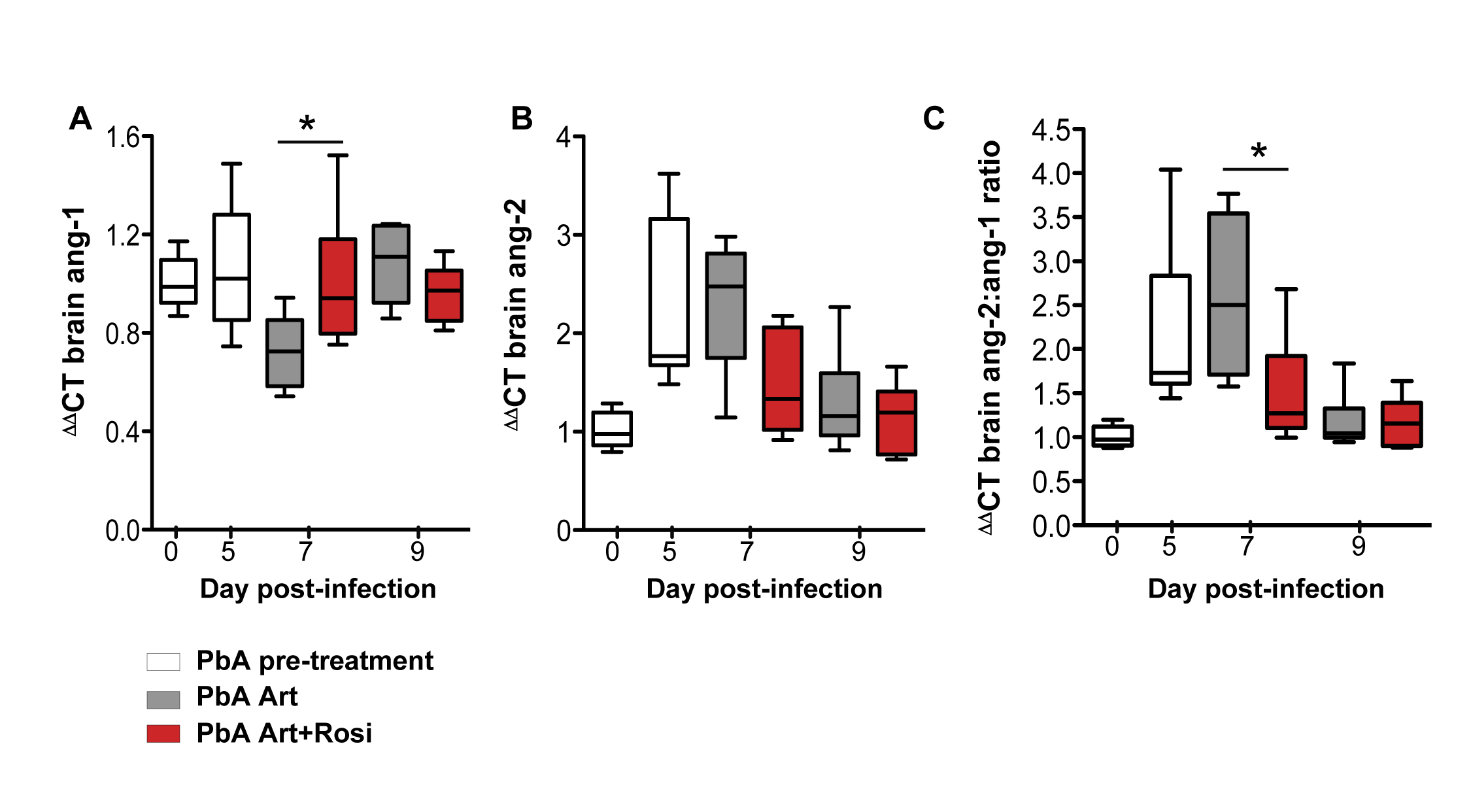

Supplement: Figure S3 — Brain expression levels of angiopoietin-1 are increased, and the ang-2 to ang-1 ratio is decreased in mice infected with P. berghei ANKA treated with rosiglitazone adjunctive therapy. Mice infected with P. berghei ANKA were treated with artesunate plus saline (grey bars), or artesunate plus rosiglitazone (red bars) starting at the onset of CM signs. Expression of ang-1 (A) and ang-2 (B) mRNA was assessed in brain homogenates collected from uninfected mice (day 0), infected mice prior to the initiation of therapy (day 5), and infected mice following treatment initiation (on day 7 and 9 post-infection). The ratio of ang-2:ang-1 was also calculated (C). Data were analysed by Kruskal-Wallis test with Dunn's post-test, N = 6/group. Significant differences were observed for ang-1 and the ang2:ang1 ratio on day 7. Ang-2 levels were lower in the rosiglitazone-treated group but did not reach significance. * p<0.05. (TIF) [file ppat.1003980.s003.tif]

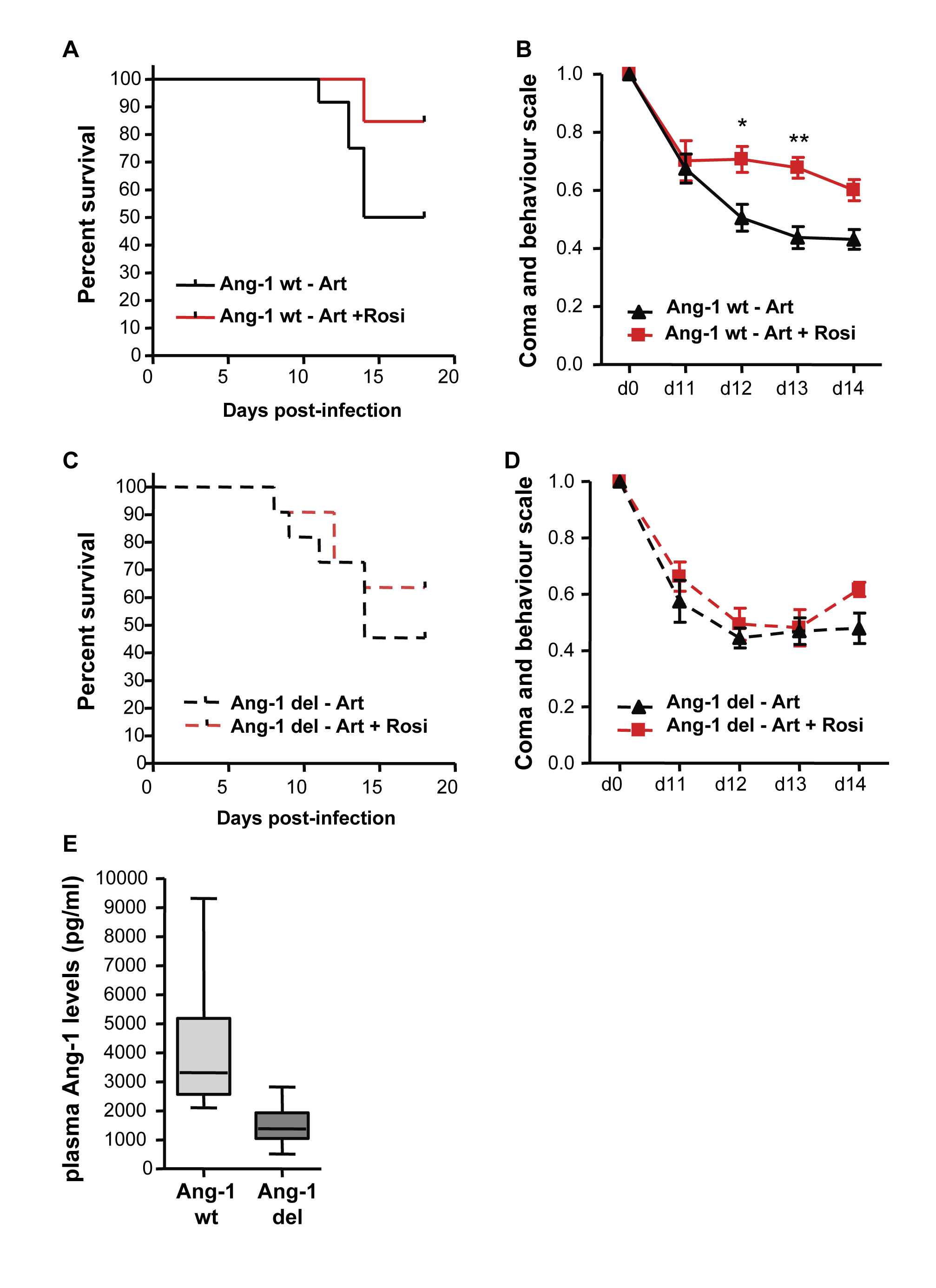

Supplement: Figure S4 — Rosiglitazone improves survival and reduces disease severity in mice sufficient in angiopoietin-1 but not in mice with an angiopoietin-1 deletion. Mice either sufficient for Ang-1 (solid lines) (A–B) or that have one Ang-1 allele deleted (Ang-1del; dashed lines) (C–D) were infected with P. berghei ANKA and treated with artesunate plus saline (Art; shown in black) or artesunate plus rosiglitazone (Art + Rosi; shown in red) starting on day 5.5 post-infection. Survival curves are shown in (A) and (C). Rosiglitazone adjunctive therapy improved survival only in the Ang-1 sufficient mice (P<0.05, by Logrank test, N = 11–15/group), but not in the Ang-1del mice. A modified coma and behavioural score was used to assess disease severity [1]. Mice were assessed on consecutive days as shown. Data for the Ang-1 sufficient mice are shown in (B) and for the Ang-1del in (D). Rosiglitazone adjunctive therapy reduced disease severity (i.e. higher score) only in the Ang-1 sufficient mice. Data are means with SEM, N = 11–15/group. Statistical comparison by two-way ANOVA with Bonferroni post-test. * P<0.05, ** P<0.01. (E) Baseline plasma levels of angiopoietin-1 in Ang-1 sufficient and Ang-1del mice. P<0.0001 by Mann Whitney test. (TIF) [file ppat.1003980.s004.tif]

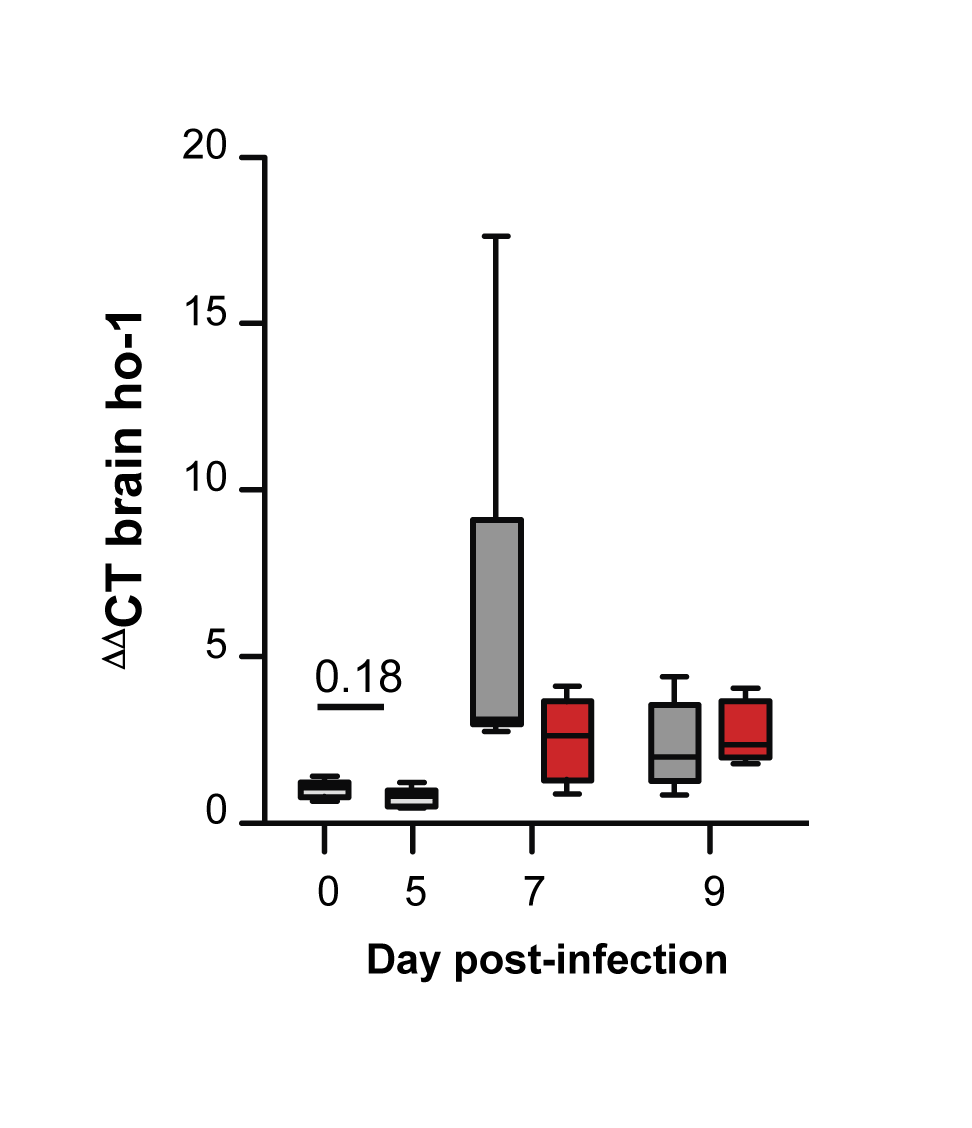

Supplement: Figure S5 — HO-1 expression levels in the brain of mice infected with P. berghei ANKA are unaffected by rosiglitazone adjunctive therapy. Mice infected with P. berghei ANKA were treated with artesunate plus saline (grey bars), or artesunate plus rosiglitazone (red bars) starting at the onset of CM signs. Expression of HO-1 mRNA was assessed in brain homogenates collected from uninfected mice (day 0), infected mice prior to the initiation of therapy (day 5), and infected mice following treatment initiation (on day 7 and 9 post-infection). Data were analysed by Kruskal-Wallis test with Dunn's post-test, N = 6 per group. (TIF) [file ppat.1003980.s005.tif]

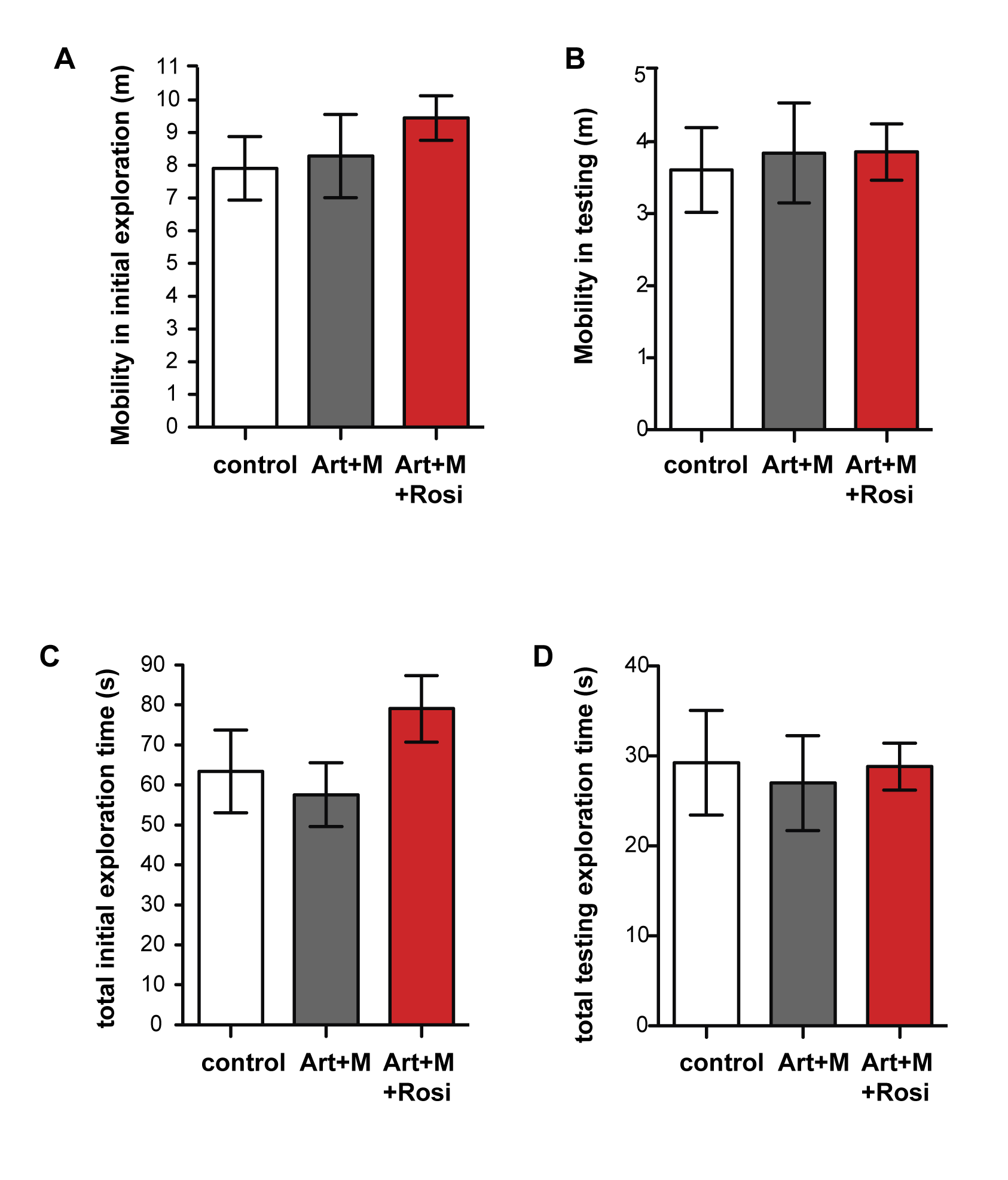

Supplement: Figure S6 — Mobility and exploration time in the novel object recognition test are similar between treatment groups. Mice infected with P. berghei ANKA were drug-cured at the onset of CM with either artesunate/mefloquine plus saline (grey bars), or artesunate/mefloquine plus rosiglitazone (red bars). Drug-treated uninfected mice were used as controls (white bars). Testing was performed 2 months following completion of treatment. Data for mobility in the initial exploration round of the NOR test are shown in (A). Data on the mobility of the mice during testing are shown in (B). Data on the total initial exploration time are shown in (C), and for the total exploration time during testing in (D). All data are means with standard deviations. No significant differences are seen between treatment groups, as assessed by one-way ANOVA. Abbreviations: Art, artesunate; M, mefloquine; Rosi, rosiglitazone. (TIF) [file ppat.1003980.s006.tif]

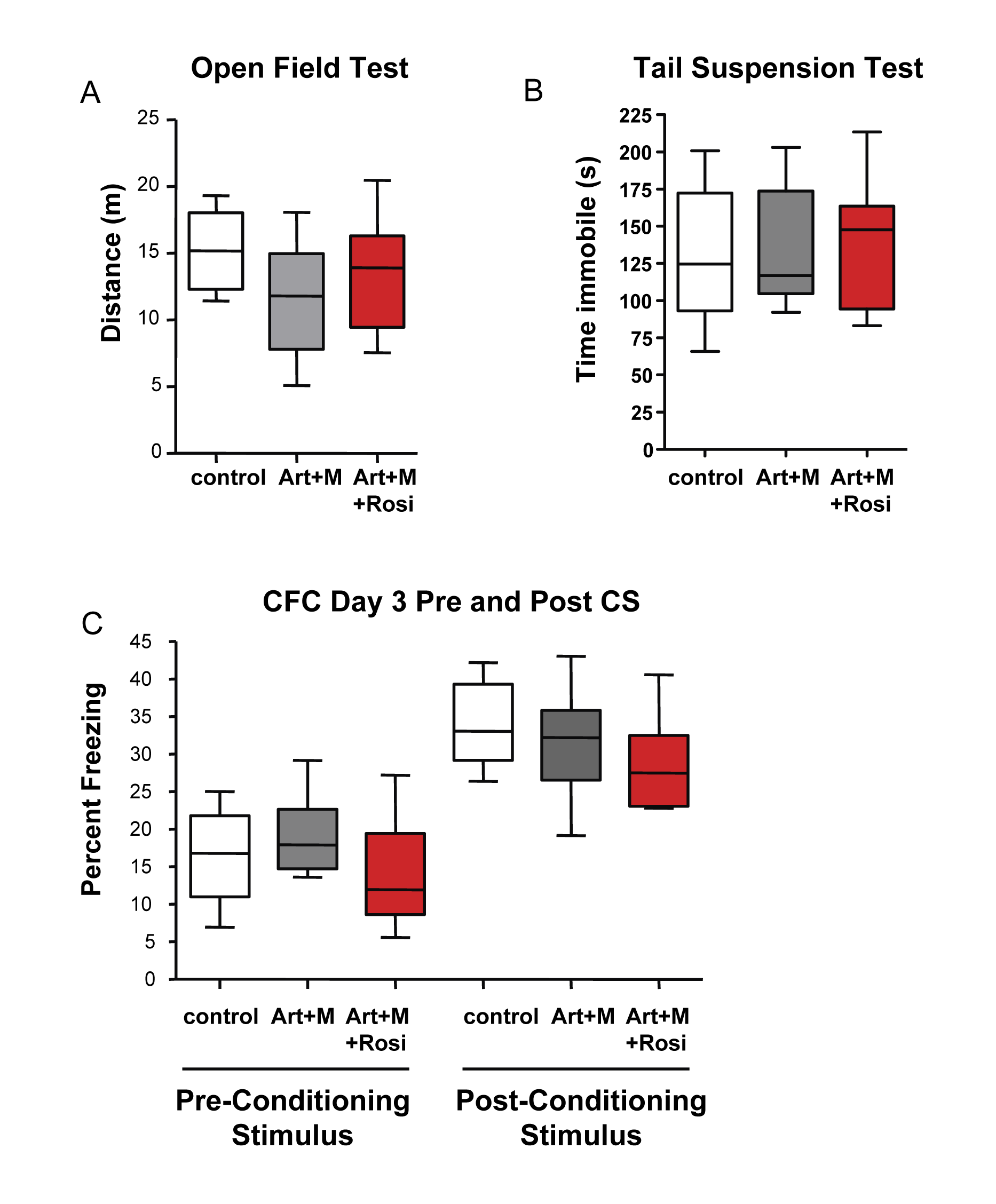

Supplement: Figure S7 — Performance in the Open Field, Tail Suspension, and Contextual Fear Conditioning Test do not differ between treatment groups. Mice infected with P. berghei ANKA were drug-cured at the onset of CM with either artesunate/mefloquine plus saline (grey bars) or artesunate/mefloquine plus rosiglitazone (red bars). Drug-treated uninfected mice were used as controls (white bars). Testing was performed 2 months following completion of treatment. Data from the Open Field test are shown in (A). Total distance covered during the Open Field Test is shown. Data from the Tail Suspension test are shown in (B). Data from the Contextual Fear Conditioning test are shown in (C). Freezing assessed prior to the conditioning stimulus and post the conditioning stimulus is shown. All data shown are medians with range. No significant differences were observed between groups as assessed by Kruskal-Wallis test. (TIF) [file ppat.1003980.s007.tif]

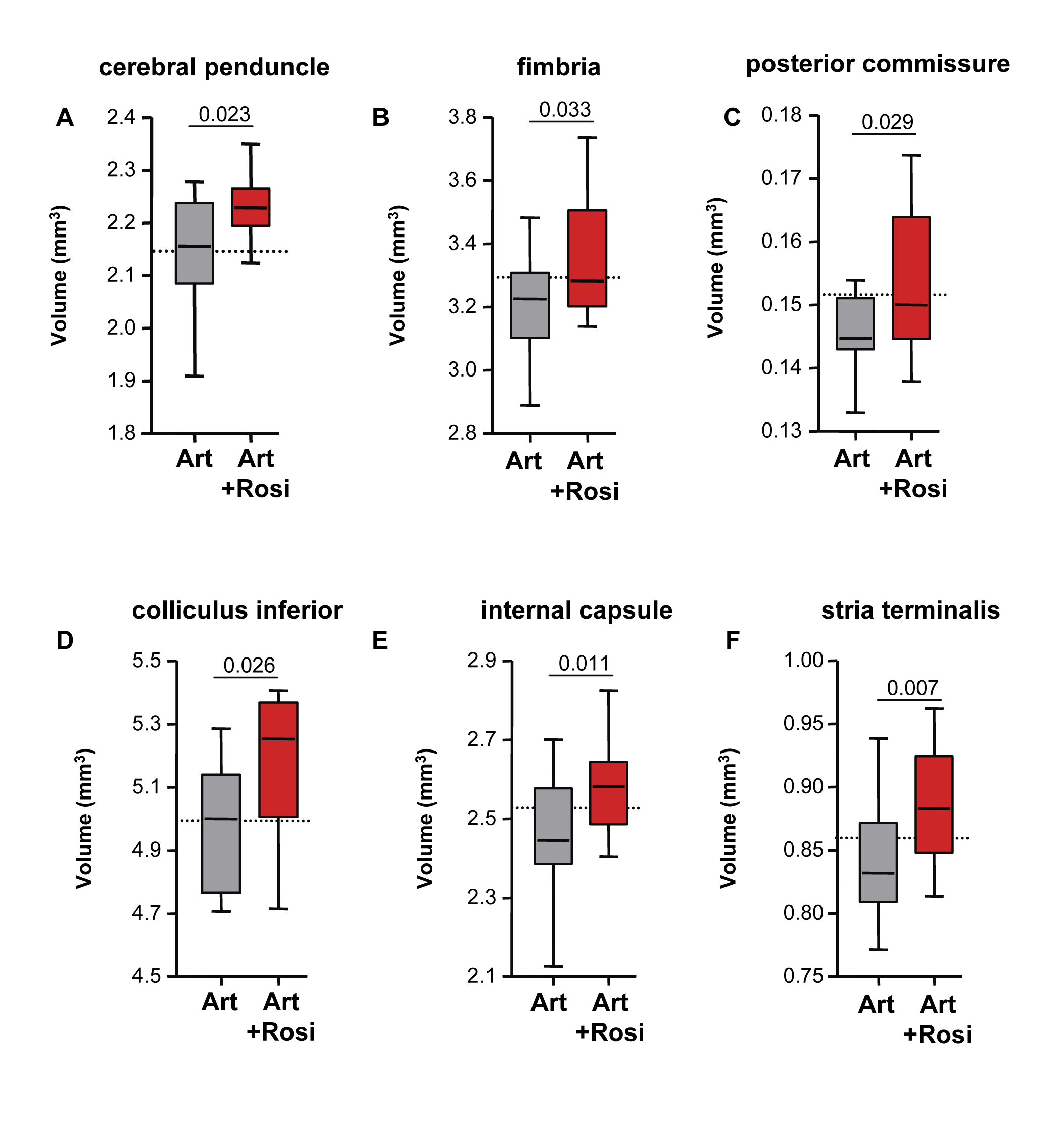

Supplement: Figure S8 — Rosiglitazone adjunctive therapy protects mice from malaria-induced brain atrophy. Following completion of all behavioural testing mice were sacrificed and their brains scanned using magnetic resonance imaging (MRI). This was followed by image registration and volumetric analysis of the brain volume of 62 distinct regions. Linear regression analysis was performed to identify areas that differed significantly between the mice treated with artesunate/mefloquine plus saline (grey bars), and the mice treated with artesunate/mefloquine plus rosiglitazone (red bars). Significant differences were observed in (A) the cerebral penduncle, (B) the fimbria, (C) the posterior commissure, (D) the colliculus inferior, (E) the internal capsule, and (F) the stria terminalis. N = 13 for artesunate/mefloquine, N = 10 for artesunate/mefloquine + rosiglitazone. The median volume for uninfected mice is shown as a dashed line (N = 10 for uninfected control). (TIF) [file ppat.1003980.s008.tif]
