# Supplementary material for: PPARγ Agonists Improve Survival and Neurocognitive Outcomes in Experimental Cerebral Malaria and Induce Neuroprotective Pathways in Human Malaria
Source: PLoS Pathog. 2014 Mar 6;10(3):e1003980. doi: 10.1371/journal.ppat.1003980 (PMC3946361; doi:10.1371/journal.ppat.1003980)
Supplement: Table S1 — Primers used for qRT-PCR analysis of brain homogenates. (DOC) [file ppat.1003980.s009.doc]

| **Gene** | **Forward Primer Sequence** | **Reverse Primer Sequence** |
| --- | --- | --- |
| Ang-1 | CCT CTG GTG AAT ATT GGC TTG GGA | AGC ATG TAC TGC CTC TGA CTG GTT |
| Ang-2 | AGA GTA CTG GCT GGG CAA TGA GTT | TTC CCA GTC CTT CAG CTG GAT CTT |
| BDNF | GCGCCCATGAAAGAAGTAAA | TTCGATGACGTGCTCAAAAG |
| NGF | CAGACCCGGAACATCACTGTA | CCATGGGCCTGGAAGTCTAG |
| Trk-B | CGCCCTGTGAGCTGAACTCTG | CTGCTTCTCAGCTGCCTGACC |
| HO-1 | CACGCATATACCCGCTACCT | CCAGAGTGTTCATTCGAGCA |
| SOD1 | AGGCTGTACCAGTGCAGGAC | GTTTACTGCGCAATCCCAAT |
| Catalase | CCTCGTTCAGGATGTGGTTT | GGCATCCCTGATGAAGAAAA |
| GAPDH | TCAACAGCAACTCCCACTCTTCCA | TTGTCATTGAGAGCAATGCCAGCC |

Table S1: Primers used for qRT-PCR analysis of brain homogenates
