# Supplementary material for: PPARγ Agonists Improve Survival and Neurocognitive Outcomes in Experimental Cerebral Malaria and Induce Neuroprotective Pathways in Human Malaria
Source: PLoS Pathog. 2014 Mar 6;10(3):e1003980. doi: 10.1371/journal.ppat.1003980 (PMC3946361; doi:10.1371/journal.ppat.1003980)
Supplement: Text S1 — Supplemental methods. (DOC) [file ppat.1003980.s010.doc]

**Supplemental Methods:**

**The Open Field Test:** The open field test was used to assess exploratory and locomotor behaviour. This is a well-validated test of normal exploratory behaviour and hyperactivity [2]. Animals were placed in a clean, empty open field (40cm x 40cm x 50cm) made of plywood and painted white with non-toxic acrylic paint. Animals freely explored the maze for 30 minutes and their behaviour was video recorded using ANYMAZE software. The following behaviours were coded: immobility, grooming, rearing, and locomotion.

**The Tail Suspension Test (TST):** The TST is a well-validated murine model of affective (anxiety-like) behaviour [3]. Animals with higher levels of anxiety-like behaviour show increased immobility (freezing) compared to animals with normal baseline levels of anxiety-like behaviour that show higher levels of mobility during testing. The testing protocol was performed as previously described [4-5]. Each mouse was suspended by a small piece of masking tape on the tail for a 6 minute duration. All tests were video recorded using ANYMAZE software. The mobility (mouse moving limbs and making active effort to escape) and immobility (freezing behaviour with no movement in body or limbs) were coded during testing.

**The Contextual Fear Conditioning (CFC) Test:** The CFC test is used to assess learning and memory dependent upon hippocampal (spatial learning) and amygdala (emotional, cued learning) function [6]. All testing was conducted using a computer-controlled fear conditioning system (TSE, Bad Homburg, Germany). Fear conditioning took place in a plexiglass chamber (20cm x 20 cm x 36 cm) within a fear-conditioning box under constant illumination. During the training and testing mice are single housed and brought into the testing room individually to prevent pre-testing exposure to the testing room and tone. The conditioning trial (Day 1) consisted of a single trial where the mouse was placed in the plexiglass chamber (conditioning context) for 180s, after which a 30s tone was played (10 kHz, 75 dB SPL). Termination of the tone coincided with onset of a 2s shock (0.7 mA, constant current) delivered through a stainless steel grid floor. The mouse was left in the plexiglass chamber for 30s so that handling upon removal from the testing chamber was not associated with shock. Contextual memory was assessed 24h following the conditioning trial (Day 2). Mice were returned to the plexiglass chamber and left for 210s. Conditioned memory was assessed 48h following completion of the conditioning trial (Day 3). Mice were again returned to the plexiglass testing chamber. The context was altered by replacing the stainless steel beam floor with a smooth plastic floor and covering the plexiglass chamber walls with paper towel. After 180s in the chamber the tone was played for 180s. Across days 1-3, total freezing (as measured by total number of beam breaks) was recorded using the fear conditioning system.

**MRI acquisition:** A multi-channel 7.0-T MRI scanner (Varian Inc., Palo Alto, CA) containing a 40cm diameter bore magnet scanned perfused brain tissue within sculls. Prior to imaging, samples were removed from solution C, blotted and placed into 13mm diameter plastic tubing filled with a proton-free susceptibility-matching solution (Florinert FC-77, 3 M Corp., St. Paul, MN). Custom built, solenoid coils were use to image multiple specimens at one time. Scan parameters were set to optimize grey/white matter contrast: T2 weighted, 3D fast spin echo sequence with TR = 2000 ms, echo train length = 6, TE_eff_ = 42 ms, field-of-view (FOV) = 25 x 28 x 14 mm and matrix size = 450 x 504 x 250, resulting in a final image with 56μm isotropic voxel. Total scan time was 11.7 hours.

**Image analysis:** Image analysis was performed as previously described [7]. Briefly, a series of registration steps employing linear and non-linear transformations was used to bring all scans into anatomical alignment in an unbiased fashion. This analysis results in a deformation field for each mouse that encodes how the mouse differs from the group average. All registrations were performed using a combination of mni_autoreg tools [8] and ANTS [9]. After registration, a pre-existing MRI anatomical atlas [10] was aligned with the group average. Using the atlas in conjunction with the deformation fields, the volume of each of 62 anatomical structures was computed for each mouse. Student *t*-tests were used to compare the anatomical volumes, controlling for multiple comparisons using the False Discovery Rate method [11].

**Supplemental references:**

1. Carroll RW, Wainwright MS, Kim KY, Kidambi T, Gomez ND, et al. (2010) A rapid murine come and behavior scale for quantitative assessment of murine cerebral malaria. PLoS One 5: e13124.
2. Belzung C, Griebel G (2001) Measuring normal and pathological anxiety-like behaviour in mice: a review. Behav Brain Res 125: 141-149.
3. Cryan JF, Holmes A (2005) The ascent of mouse: advances in modeling human depression and anxiety. Nat Rev Drug Discov 4: 775-790.
4. Genoux D, Haditsch U, Knobloch M, Michalon A, Storm D, et al. (2002) Protein phosphatase 1 is a molecular constraint on learning and memory. Nature 29: 970-975.
5. Francis BM, Yang J, Hajderi E, Brown ME, Michalski B, et al. (2012) Reduced tissue levels of noradrenaline are associated with behavioral phenotypes of the TgCRND8 mouse model of Alzheimer’s disease. Neuropsychopharmacology 37: 1934-1944.
6. Falls WA (2002) Fear-potentiated startle in mice. Curr Protoc Neurosci chapter 8, Unit 8.11b.
7. Lerch JP, Sled JG, Henkelman RM (2011) MRI phenotyping of genetically altered mice. Methods Mol Biol 711: 349-361.
8. Collins DL, Neelin P, Peters TM, Evans AC (1994) Automatic 3D intersubject registration of MR volumetric data in standardized talairach space. J Comput Assist Tomogr 18: 192–205.
9. Avants BB, Yushkevich P, Pluta J, Minkoff D, Korczykowski M, et al. (2010) The optimal template effect in hippocampus studies of diseased populations. NeuroImage 49: 2457–2466.
10. Dorr AE, Lerch JP, Spring S, Kabani N, Henkelman RM (2008) High resolution three-dimensional brain atlas using an average magnetic resonance image of 40 adult C57Bl/6J mice. Neuroimage 42: 60-69.
11. Genovese CR, Lazar NA, Nichols T (2002) Thresholding of statistical maps in functional neuroimaging using the false discovery rate. NeuroImage 15: 870–878.
